# Factor price distortion among regions in China and its influence on China’s economic growth

**DOI:** 10.1371/journal.pone.0284191

**Published:** 2023-04-10

**Authors:** JIE Yang

**Affiliations:** School of Finance, Guangdong University of Foreign Studies, Guangzhou, Guangdong Province, PR China; University of Pisa, ITALY

## Abstract

Factor price distortions and resource misallocation are important sources of productivity differences between regions. Promoting the free flow of factors of production is conducive to giving full play to the decisive role of the market in allocating resources, which is crucial to helping a country’s economy develop in a high-quality and sustainable manner. This paper proposes a new approach to measuring factor market distortions and establishes the relationship between factor price distortions and a country’s economic growth. This paper examines the resource misallocation and efficiency loss of 31 provinces in China from 2004 to 2020, and proposes an analytical framework for resource misallocation among regions, with which the Total Factor Productivity (TFP) and the factor price distortion of provinces in China are calculated. The calculation results indicate that the TFP of China’s provinces gradually declines from the eastern coast to the western inland. The resource allocation efficiency in the eastern and central areas is higher than that in the western areas, so is the factor price, and its distortion causes nearly 6% of loss of output value in China. China’s economic growth is still reliant on the increase of factor input and technological development and the improvement of resource allocation efficiency has no significant effect on growth.

## Introduction

Since the reform and opening-up, China’s economy has experienced unprecedented growth. In 1980, the initial stage of the reform and opening-up, China’s per capita gross domestic product (GDP) was only 194.8 US dollars. After 40 years of high-speed growth, it reached 12,556.3 US dollars in 2021, more than 60 times that of 1980. However, the growth rate of China’s per capita GDP has continued to decline since the 2008 financial crisis. In 2007, a year before the financial crisis, the growth rate hit a whopping 13.64%, but it dropped to 9.90% in 2008 and continued to plummet into 6.42% in 2015. As of 2021, the growth rate was 8.00%, still lower than the pre-crisis level. People began to worry about the long-term sustainability of China’s rapid economic growth and try to explore new growth engines. To address these issues, this paper seeks to examine whether economic growth can be driven by improving China’s resource misallocation situation. This paper uses a new methodology to measure the extent of resource misallocation in China and establishes a general relationship between factor price distortions and economic growth.

Many scholars have started to search for new growth points for China, and their studies focus on improving resource allocation efficiency and increasing TFP [1–3]. Although by investing a large amount of resources, China achieved economic growth in the initial stage of reform and opening-up, the resource input in China has been enormous and the cost of production factor has risen [4,5], low fertility implies a shortage of labor and hinders productivity growth in China in the future [6]. The increase of production factor supply fails to meet the demand of sustained rapid economic growth. Chinese government believes that it is no longer viable for China to rely on an extensive growth mode that boosts economic growth by increasing factor input. On the contrary, China should pursue an intensive growth mode with high TFP depending on technology advances, organizational innovation, specialization and production innovation [7,8]. Therefore, it is necessary to improve resource allocation efficiency and TFP to realize economic growth with no or little increase in factor input. Hsieh and Klenow [9] found that if the resource allocation efficiency of China or India was improved to the level of the US, their TFP will increase by 30%-50% and 40%-60% respectively, bringing a considerable output growth. Dollar and Wei [10] argued that the resource allocation in Chinese enterprises is inefficient, with low-productivity enterprises occupying a disproportionate amount of production factors. If those factors were transferred to high-productivity enterprises, China’s output and TFP will both grow by nearly 40%. Therefore, resource allocation efficiency has a significant impact on China’s economic development. Measuring China’s resource misallocation and optimizing its resource allocation efficiency are important driving forces to revive China’s economy.

This paper attempts to quantitatively measure the resource misallocation among regions in China and the resulted output losses. We constructed a production model of a country with factor price distortion, and assumed that the provinces in this country face different factor prices and compete for production factors. Based on this model, this paper estimates the production function and TFP of 31 provinces in Chinese mainland. The research result displays that China’s economic production is highly dependent on capital as its contribution to output is about 55% while that of labor and land are 25% and 20% respectively. Moreover, there is a huge discrepancy in the TFP of different provinces in China. The TFP of the eastern coastal provinces is markedly higher than that of the provinces in the central and western areas. By calculating the relative distortion coefficients of production factors, it is found that resources in western China are over-allocated, while those in eastern and central China are under-allocated. This paper holds that this is resulted from government interference, which provides the western provinces with an access to more and cheaper production factors. In the end, production factor misallocation leads to an output loss of 6% in China, but the factor price distortion is aggravating in China in the recent decade. China’s economic growth is mainly reliant on the increase of factor input and TFP while the improvement of factor allocation efficiency has no significant effect on economic growth.

The contributions of this paper are as follows. Firstly, a model for measuring the efficiency of macro resource allocation is constructed, which can be applied to comparing the resource allocation among regions in a country. Existing studies on China’s resource misallocation concentrate on its cause [11,12] and the efficiency loss caused [13,14]. However, none of these studies has conducted quantitative measurement of the resource allocation efficiency of regions in China so that the spatial resource misallocation in China cannot be observed from a macro perspective. Hence, the model proposed in this paper will provide a quantitative framework for subsequent studies. Secondly, this paper uses the data of Chinese cities to study the TFP and resource misallocation of provinces in China. Most previous studies of resource misallocation utilize enterprise data to measure the TFP and resource misallocation of industries [9,15,16]. These studies measure the allocation efficiency of production factors among industries and then evaluate resource allocation efficiency and industrial structure of a country. They are all conducted from an industrial perspective. However, they lack a regional perspective. To fill this gap, this paper analyzes China’s spatial resource misallocation using the data of Chinese cities. Thirdly, we take land into account as the third production factor except capital and labor in the research of resource misallocation. Most studies are known to take only capital and labor into account as production factors [9,17,18]. But we consider land as a significant production factor in China as well. The reasons are as follows. One is that China’s economic growth has long relied on the increase of both the land supply for industrial use and the urban built-up areas, thus, land allocation efficiency is highly correlated to economic fluctuation [19]. The other is that land input has greatly promoted regional economic development in China, particularly in the Primary Production Stage (PPS) and the Primary Industrialization Stage (PIS) of cities [20].

The rest of the paper is organized as follows. The section 2 provides a literature review on resource misallocation and factor market distortions. In Section 3, we present the theoretical framework for measuring factor misallocation and output losses. In Section 4, we discuss the data used for empirical analysis and present the analysis result. We also discuss and express our opinions on the major results in this section. Finally, conclusions are presented in Section 5.

## Literature review

Distortions in the prices of factors of production such as capital, labor, and land can arise due to, for example, government intervention and restrictions on factor mobility [21–23]. The distortion of factor prices can further deviate the allocation of factors of production between different enterprises or different regions from the optimal state, that is resource misallocation [9]. Resource misallocation studies the gap between the actual allocation of resources and the efficient allocation. An efficient allocation represents the maximum output that an economy can create with its limited resources.

There are two research frameworks for resource misallocation, the direct approach and the indirect approach [24]. The direct approach is a local analysis, and the indirect approach is a general analysis. The direct approach focuses on a specific factor that causes the misallocation and analyses the loss of output due to this factor. For example, Hopenhayn and Rogerson [25] studied the distortion of labor allocation by worker termination taxes, Bai et al. [21] studied the effect of government subsidies and tax incentives on factor market distortions, and Bai et al. [22] found that politically connected firms are more likely to receive special favors from the government. In contrast, unlike the direct approach, the indirect approach does not analyze the causes of misallocation and only measures the difference in output value between actual and efficient resource allocation. The indirect method examines the additive impact of all factors and does not focus on one particular factor. Representative studies include Hsieh and Klenow [9], who find that without factor market distortions, China’s output levels would have increased by 86.6–115.1% between 1998 and 2005, and Brandt et al. [15] found that resource misallocation reduced China’s non-agricultural TFP between 1985 and 2007 by about 20%. In order to take a perspective of the whole, this paper will use an indirect approach to measure resource misallocation between regions in China.

Regional resource misallocation, also called spatial resource misallocation, refers to the non-optimal allocation of resources between regions in proportion to each other. Regional resource allocation not only studies the distribution of production factors between different regions but also the circulation of production factors between regions. Regional resource allocation has a significant impact on regional economic development and the income levels of residents, as well as on the development gap between regions [15]. Current research on the issue in China has focused on the scale and direction of factor flows between regions [26], as well as the mechanisms and impacts of factor misallocation [12,14,20]. However, none of these studies have measured resource misallocation between regions in China over the last decade, and lack an overall analysis of the current spatial misallocation in China. Moreover, no literature has systematically examined how resource misallocation in China has affected economic development. Therefore, this paper constructs an accounting model to measure the efficiency of resource allocation between different regions and then uses this model to evaluate the allocation efficiency of China’s provinces. In order to examine more intuitively the impact of resource misallocation on economic development, this paper creatively proposes a quantitative model to measure factor market distortions and economic growth rates.

## Theoretical model

### Fundamental assumption

We posit that there is a country that consists of *n* provinces and study the factor misallocation among provinces in this country. It is assumed that each province competes for the limited production factors, but factor prices vary among provinces. In consequence, we constructed a production model of the country with factor price distortion. We will first define the absolute distortion coefficient and the relative distortion coefficient of factors and then calculate the relative distortion coefficient and output on the premise that production factors maintain perfect competitive equilibrium.

### Production functions of a country containing n provinces

Given that our study focuses on the resource misallocation among provinces in a country, we assume that the production functions of all manufacturers in a province are the same, and the production in each province is conducted by one representative manufacturer. In most cases, the production functions differ among provinces.

The paper considers three production factors, capital (*K*), labor (*L*) and land (*M*). All provinces use the three factors for production and are assumed as price taker. Assuming that there exists production factor distortion, the degree of distortion is presented in the form of ad valorem tax (*τ*), which varies from province to province. Hence, the prices of the three production factors province *i* faces are (1 + *τ*_*Ki*_)*P*_*K*_, (1 + *τ*_*Li*_)*P*_*L*_, (1 + *τ*_*Mi*_)*P*_*M*_. *P*_*K*_, *P*_*L*_, *P*_*M*_ each represents a factor price without distortion and *τ*_*Ki*_, *τ*_*Li*_, *τ*_*Mi*_ are the distorted tax rates of factors of province *i* respectively. Suppose the production function of province *i* is Cobb-Douglas production function:

$$Y_{i}=TFP_{i}\cdot K_{i}{}^{\beta_{Ki}}L_{i}{}^{\beta_{Li}}M_{i}{}^{\beta_{Mi}}$$ (1)

where *Y*_*i*_ represents the gross output value of province *i*, *K*_*i*_, *L*_*i*_, *M*_*i*_ each represents the amount of factor input of capital, labor and land, and *β*_*Ki*_, *β*_*Li*_, *β*_*Mi*_ are the output elasticity of the three factors respectively.

The production function of the country can be obtained by summing up the production functions of all provinces. Postulating that the country’s price index (*P*) is equivalent to 1, its gross output *Y* is the linear function of the output of all provinces:

$$Y=\sum_{i=1}^{N}P_{i}Y_{i}$$ (2)

For Eq (2), it is worth noting that a country’s economic output is equal to the sum of the output of all provinces. However, due to differences in price levels, a country’s real economic output cannot be directly summed by the nominal output of each province. Therefore, it is necessary to multiply the nominal output of individual provinces by the price index to calculate the nominal output of each province and then add them together.

It is assumed that in a given period, the national supply of production factors is an exogenous variable, i.e. the total amount of production factors can be regarded as fixed.


$$K=\sum_{i=1}^{N}K_{i},\;L=\sum_{i=1}^{N}L_{i},\;M=\sum_{i=1}^{N}{\text{M}}_{i}$$ (3)

### Distortion coefficient

In this section, we will define the two kinds of distortion coefficients used to evaluate the distortion of production factor allocation.

**Definition 1** The absolute distortion coefficient of a production factor of province *i* is equal to the ratio of the factor price without distortion to the factor price with distortion. Take capital *K* as an example, its absolute distortion coefficient is:

$$\gamma_{Ki}=\frac{P_{Ki}}{(1+\tau_{Ki})P_{Ki}}=\frac{1}{1+\tau_{Ki}}$$ (4)

Likewise, we can calculate the absolute distortion coefficients of *L* and *M* of province *i*, which represent labor and the area of land respectively. This coefficient reflects the factor price distortion caused by the distorted tax rate *τ* which makes the actual factor usage of manufacturers deviate from the optimal. If there is no distortion, that is, the distortion tax rate *τ* = 0, then the absolute distortion coefficient *γ* = 1, shows that the current actual factor price is equal to the factor price without distortion. If there exists positive distortion, that is, the distorted tax rate *τ* > 0, the absolute distortion coefficient *γ* < 1, reflects government taxation on this production factor, making the current actual factor price higher than the factor price without distortion. If there exists negative distortion, that is, the distorted tax rate *τ* < 0, the absolute distortion coefficient *γ* > 1, reflects government subsidies for the production factor, making the current actual factor price lower than the factor price without distortion.

**Definition 2** In this part, we define the relative distortion coefficient. The absolute distortion coefficient reflects the absolute level of factor allocation distortion, causing incomparability in distortion between different provinces, so the indicator should be redefined. Take capital *K* as an example again, its absolute distortion coefficient is:

$${\tilde{\gamma }}_{Ki}=\frac{\gamma_{Ki}}{\sum_{j=1}^{N}\frac{s_{j}\beta_{Kj}}{{\bar{\beta }}_{K}}\gamma_{Kj}}$$ (5)

where *s*_*j*_ is province *j*’s share of output of the whole country, namely, *s*_*j*_ = *P*_*i*_*Y*_*i*_/*Y*, and ${\bar{\beta }}_{K}$ is the weighted average of the country’s elasticity of capital output, namely, ${\bar{\beta }}_{K}=\sum_{i=1}^{N}s_{i}\beta_{Ki}$. We can compare the relative distortion coefficients of different provinces that reflects the relative level of factor prices of a certain province compared with that of other provinces. Compared with the average of the country, when ${\tilde{\gamma }}_{Ki}>1$, the capital price of province *i* is relatively low and capital is overused. When ${\tilde{\gamma }}_{Ki}<1$, the capital price of province *i* is relatively high and capital is underused. When ${\tilde{\gamma }}_{Ki}=1$, the capital price of province *i* is equal to the average of the country.

Notably, the absolute distortion coefficient *γ* will change correspondingly to the degree of change in the tax rate of production factors of each province (1 + *τ*), but the relative distortion coefficient $\tilde{\gamma }$ of all provinces remains unchanged.

#### Competitive equilibrium

Based on the above models, we can work out the balanced use of factors of all provinces in the presence of factor price distortion and calculate the relative distortion coefficients of factors in each province.

**Proposition 1** It is presumed that the distorted tax rates of factors of different provinces *τ*_*Ki*_, *τ*_*Li*_, *τ*_*Mi*_ and the total resources of the country *K*, *L*, *M* are given. On the premise of competitive equilibrium, every province realizes the maximization of profit (*π*):

$$\underset{Ki,Li,Mi}{\text{max}}\;\pi_{i}=P_{i}Y_{i}-(1+\tau_{Ki})P_{K}K_{i}-(1+\tau_{Li})P_{L}L_{i}-(1+\tau_{Mi})P_{M}M_{i}$$ (6)

where *P*_*i*_ represents the price index of province *i*. By calculating the partial derivative of Eq (6), we can obtain:

$$\left\{\begin{array}{l} \frac{\partial \pi_{i}}{\partial K_{i}}=0\\ \frac{\partial \pi_{i}}{\partial L_{i}}=0\\ \frac{\partial \pi_{i}}{\partial M_{i}}=0 \end{array}\right.\Rightarrow \left\{\begin{array}{l} \beta_{Ki}P_{i}\cdot TFP_{i}\cdot K_{i}^{\beta_{Ki}-1}L_{i}^{\beta_{Li}}M_{i}^{\beta_{Mi}}-(1+\tau_{Ki})P_{K}=0\\ \beta_{Li}P_{i}\cdot TFP_{i}\cdot K_{i}^{\beta_{Ki}}L_{i}^{\beta_{Li}-1}M_{i}^{\beta_{Mi}}-(1+\tau_{Li})P_{L}=0\\ \beta_{Mi}P_{i}\cdot TFP_{i}\cdot K_{i}^{\beta_{Ki}}L_{i}^{\beta_{Li}}M_{i}^{\beta_{Mi}-1}-(1+\tau_{Mi})P_{M}=0 \end{array}\right.$$ (7)

As the total amount of resources of the country is fixed, combining Eqs (3) and (7), *K*_*i*_, *L*_*i*_, *M*_*i*_ of each province can be obtained:

$$K_{i}=\frac{\frac{\beta_{Ki}P_{i}Y_{i}}{(1+\tau_{Ki})P_{K}}}{\sum_{j=1}^{N}\frac{\beta_{Kj}P_{j}Y}{(1+\tau_{Kj})P_{K}}}K,\;L_{i}=\frac{\frac{\beta_{Li}P_{i}Y_{i}}{(1+\tau_{Li})P_{L}}}{\sum_{j=1}^{N}\frac{\beta_{Lj}P_{j}Y}{(1+\tau_{Lj})P_{L}}}L,\;\;M_{i}=\frac{\frac{\beta_{Mi}P_{i}Y_{i}}{(1+\tau_{Mi})P_{M}}}{\sum_{j=1}^{N}\frac{\beta_{Mj}P_{j}Y}{(1+\tau_{Mj})P_{M}}}M$$ (8)

The economic implication of Eq (8) is easy to understand. Take capital *K*_*i*_ as an example: *β*_*Ki*_*P*_*i*_*Y* is the contribution of capital *K* to the output of province *i*, that is, the returns that province *i* should pay to the capital *K*. (1 + *τ*_*Ki*_)*P*_*K*_ is the cost of capital *K* in province *i*. Therefore, the numerator on the right side of the equation is the usage of capital *K* in province *i*. The denominator is the sum of capital *K* use of all provinces, or the total capital amount of the country.

**Proposition 2** In this part, we will derive a equation for the relative distortion coefficient that will be applied to empirical analysis. If the distorted tax rate *τ* in Eq (8) is replaced by the relative distortion efficient $\tilde{\gamma }$, combining (5) and (8), we can obtain:

$$K_{i}=\frac{s_{i}\beta_{Ki}}{{\bar{\beta }}_{K}}{\tilde{\gamma }}_{Ki}K,\;\;L_{i}=\frac{s_{i}\beta_{Li}}{{\bar{\beta }}_{L}}{\tilde{\gamma }}_{Li}L,\;M_{i}=\frac{s_{i}\beta_{Mi}}{{\bar{\beta }}_{M}}{\tilde{\gamma }}_{Mi}M$$ (9)

The specific derivation of Eq (9) is presented in Appendix 1.

Extract the relative distortion factor $\tilde{\gamma }$ to the left side of the equation:

$${\tilde{\gamma }}_{Ki}=\left(\frac{K_{i}}{K}\right)/\left(\frac{s_{i}\beta_{Ki}}{{\bar{\beta }}_{K}}\right),\;{\tilde{\gamma }}_{Li}=\left(\frac{L_{i}}{L}\right)/\left(\frac{s_{i}\beta_{Li}}{{\bar{\beta }}_{L}}\right),\;\;{\tilde{\gamma }}_{Mi}=\left(\frac{M_{i}}{M}\right)/\left(\frac{s_{i}\beta_{Mi}}{{\bar{\beta }}_{M}}\right)$$ (10)

The economic implication of Eq (10) is also easy to understand. Take the relative distortion efficient of capital ${\tilde{\gamma }}_{Ki}$ as an example: *K*_*i*_/*K* is the actual share of the capital use *K*_*i*_ of province *i* in this country with a distorted competitive equilibrium; $s_{i}\beta_{Ki}/{\bar{\beta }}_{K}$ is the theoretical share of the capital use *K*_*i*_ of province *i* in this country without a distorted competitive equilibrium. Eq (10) intuitively reveals that the relative distortion coefficient $\tilde{\gamma }$ reflects a province’s overuse or underuse of production factors with distortion.

### Decomposition of output

In the previous section, we calculate the relative distortion coefficient under competitive equilibrium. This indicator can reflect the relative surplus or deficiency of factor allocation among provinces, but it cannot reflect the impact of factor misallocation on the output of the whole country. In order to fill this gap, this study attempts to decompose the country’s production functions to find out the motivators for economic growth. We first decompose the production function of each province, and then add up the results of the decomposition equations of all provinces to get the decomposition equation of the whole country.

#### Decomposition of provincial output

Put Eq (9) into (1), we can obtain:

$$\begin{array}{ll} Y_{i} & =TFP_{i}\cdot K_{i}{}^{\beta_{Ki}}L_{i}{}^{\beta_{Li}}M_{i}{}^{\beta_{Mi}}\\ & =TFP_{i}\cdot {\left(\frac{s_{i}\beta_{Ki}}{{\bar{\beta }}_{K}}{\tilde{\gamma }}_{Ki}K\right)}^{\beta_{Ki}}{\left(\frac{s_{i}\beta_{Li}}{{\bar{\beta }}_{L}}{\tilde{\gamma }}_{Li}L\right)}^{\beta_{Li}}{\left(\frac{s_{i}\beta_{Mi}}{{\bar{\beta }}_{M}}{\tilde{\gamma }}_{Mi}M\right)}^{\beta_{Mi}} \end{array}$$ (11)

and take the log of the both sides of Eq (11)

$$\begin{array}{ll} \text{ln}\;Y_{i} & =\underset{A}{\underset{︸}{\text{ln}\;TFP}}+\underset{B}{\underset{︸}{\text{ln}\left(s_{i}{\left(\frac{\beta_{Ki}}{{\bar{\beta }}_{K}}\right)}^{\beta_{Ki}}{\left(\frac{\beta_{Li}}{{\bar{\beta }}_{L}}\right)}^{\beta_{Li}}{\left(\frac{\beta_{Mi}}{{\bar{\beta }}_{M}}\right)}^{\beta_{Mi}}\right)}}\\ & \text{+}\underset{C}{\underset{︸}{\left[\beta_{Ki}\;\text{ln}\left({\tilde{\gamma }}_{Ki}\right)+\beta_{Li}\;\text{ln}\left({\tilde{\gamma }}_{Li}\right)+\beta_{Mi}\;\text{ln}\left({\tilde{\gamma }}_{Mi}\right)\right]}}\\ & \text{+}\underset{D}{\underset{︸}{\left[\beta_{Ki}\;\text{ln}\;K+\beta_{Li}\;\text{ln}\;L+\beta_{Mi}M\right]}} \end{array}$$ (12)

Then, the production output ln *Y*_*i*_ is decomposed into four parts: the TFP of province *i* (A), the factor output elasticity of province *i* (B), the relative distortion coefficient of factors (C) and the factor usage (D). Hence, the production output in a certain period can be attributed to the above four aspects. Part C indicates that if the relative distortion coefficient of factors changes, or the factor allocation of a province changes, the province’s production output will change accordingly.

From Eq (12), the change of production output of province *i* from *t* to *t*+1 can be obtained.


$$\begin{array}{ll} \Delta \;\text{ln}\;Y_{it} & =\text{ln}\;Y_{it+1}-\text{ln}\;Y_{it}\\ & =\Delta \;\text{ln}\;TFP_{i}+\text{ln}[\left(\frac{s_{it+1}}{s_{it}}\right)/\left(\frac{{\bar{\beta }}_{Kt+1}^{\beta_{Ki}}{\bar{\beta }}_{Lt+1}^{\beta_{Li}}{\bar{\beta }}_{Mt+1}^{\beta_{Mi}}}{{\bar{\beta }}_{Kt}^{\beta_{Ki}}{\bar{\beta }}_{Lt}^{\beta_{Li}}{\bar{\beta }}_{Mt}^{\beta_{Mi}}}\right)]\\ & +\left[\beta_{Ki}\Delta \;\text{ln}\;{\tilde{\gamma }}_{Ki}+\beta_{Li}\Delta \;\text{ln}\;{\tilde{\gamma }}_{Li}+\beta_{Mi}\Delta \;\text{ln}\;{\tilde{\gamma }}_{Mi}\right]\\ & +\left[\beta_{Ki}\Delta \;\text{ln}\;K+\beta_{Li}\Delta \;\text{ln}\;L+\beta_{Mi}\Delta \;\text{ln}\;M\right] \end{array}$$ (13)

#### Motivators for economic growth

Based on Eq (12), the variation in the production output of the country is decomposed referring to the practice of Syrquin [27] and Chen [16]. Syrquin [27] attributed the variation of the production output of a country to TFP Growth (TFPG) and the change in the amount of factors available in the whole country. TFPG can be further divided into two parts: the TFPG of each province, and the variation of production factor allocation. On this basis, Chen [16] further decomposed the above variations of production factor allocation into the variation of factor output elasticity and that of factor price distortion. The detailed derivation is as follows:

Suppose the country’s production output reaches competitive equilibrium in each period, the variation in production output from *t* to *t*+1 is Δ ln *Y*_*t*_ = ln *Y*_*t*+1_ − ln *Y*_*t*_ and Δ ln *Y*_*t*_ can be decomposed into:

$$\begin{array}{cc} \Delta \;\text{ln}\;Y_{t} & =\text{ln}\;Y_{t+1}-\text{ln}\;Y_{t}\\ & =\sum_{i=1}^{N}s_{it}\Delta \;\text{ln}\;Y_{it}\\ & =\underset{\text{A}}{\underset{︸}{\sum_{i=1}^{N}s_{it}\Delta \;\text{ln}\;TFP_{i}}}+\underset{\text{B}}{\underset{︸}{\sum_{i=1}^{N}s_{it}\;\text{ln}[\left(\frac{s_{it+1}}{s_{it}}\right)/\left(\frac{{\bar{\beta }}_{Kt+1}^{\beta_{Ki}}{\bar{\beta }}_{Lt+1}^{\beta_{Li}}{\bar{\beta }}_{Mt+1}^{\beta_{Mi}}}{{\bar{\beta }}_{Kt}^{\beta_{Ki}}{\bar{\beta }}_{Lt}^{\beta_{Li}}{\bar{\beta }}_{Mt}^{\beta_{Mi}}}\right)]}}\\ & +\underset{\text{C}}{\underset{︸}{\sum_{i=1}^{N}s_{it}\left[\beta_{Ki}\Delta \;\text{ln}\;{\tilde{\gamma }}_{Ki}+\beta_{Li}\Delta \;\text{ln}\;{\tilde{\gamma }}_{Li}+\beta_{Mi}\Delta \;\text{ln}\;{\tilde{\gamma }}_{Mi}\right]}}\\ & +\underset{\text{D}}{\underset{︸}{\sum_{i=1}^{N}s_{it}\left[\beta_{Ki}\Delta \;\text{ln}\;K+\beta_{Li}\Delta \;\text{ln}\;L+\beta_{Mi}\Delta \;\text{ln}\;M\right]}} \end{array}$$ (14)

The derivation of Eq (14) is presented in Appendix 2. From Eq (14), we can also conclude four reasons for the variation of the country’s gross output: the sum of TFPG of all provinces (A), the variation in resource allocation of all provinces (B&C) and the factors that are available for the whole society (D). B&C can be subdivided into the elasticity variation of factor output within provinces (B) and the variation of relative distortion of factors among provinces (C).

### Effective output and output gap

B and C in Eq (14) indicate that production output is strongly linked to resource allocation, meaning that resource allocation efficiency directly influences production output. As a result, the loss of output caused by resource misallocation should be worked out in the following sections.

We presume that the production function of the country is Cobb-Douglas production function:

$$Y=\prod_{i=1}^{N}Y_{i}{}^{s_{i}}=\prod_{i=1}^{N}{\left\{TFP_{i}\cdot K_{i}^{\beta_{Ki}}L_{i}^{\beta_{Li}}M_{i}^{\beta_{Mi}}\right\}}^{s_{i}}\;\text{s}.\text{t}.\;\sum_{i=1}^{N}s_{i}=1$$ (15)

The derivation of Eq (15) is showed in Appendix 3.

**Definition 3** We suppose that each province in the country has the same factor price, meaning that there is no factor price distortion. Under perfect competitive equilibrium, the output *Y*_*i*_ of province *i* is defined as effective output, written as $Y_{i}^{eff}$. Without distortion, the absolute distortion coefficient *γ* and relative distortion coefficient $\tilde{\gamma }$ of factors are both equal to 1.

The effective output of province *i* can be obtained as follows:

$$Y_{i}^{eff}=TFP_{i}\cdot {\left(\frac{s_{i}\beta_{Ki}}{{\bar{\beta }}_{K}}K\right)}^{\beta_{Ki}}{\left(\frac{s_{i}\beta_{Li}}{{\bar{\beta }}_{L}}L\right)}^{\beta_{Li}}{\left(\frac{s_{i}\beta_{Mi}}{{\bar{\beta }}_{M}}M\right)}^{\beta_{Mi}}$$ (16)

The effective output of the entire country is:

$$Y^{eff}=\prod_{i=1}^{N}{\left(Y_{i}^{eff}\right)}^{s_{i}}=\prod_{i=1}^{N}\left\{TFP_{i}\cdot {\left(\frac{s_{i}\beta_{Ki}}{{\bar{\beta }}_{K}}K\right)}^{\beta_{Ki}}{\left(\frac{s_{i}\beta_{Li}}{{\bar{\beta }}_{L}}L\right)}^{\beta_{Li}}{\left(\frac{s_{i}\beta_{Mi}}{{\bar{\beta }}_{M}}M\right)}^{\beta_{Mi}}\right\}$$ (17)

**Definition 4** On the basis of effective output, we define the output gap *E*. It is equal to the difference between actual output and effective output divided by the effective output. It measures the loss of output due to factor price distortion. The output gap *E* is defined as:

$$E=\frac{Y^{eff}-Y}{Y^{eff}}=1-\frac{Y}{Y^{eff}}$$ (18)

According to its definition, we can calculate the output gap *E*_*i*_ of province *i*:

Using the result of Eq (9), we can obtain:

$$\begin{array}{ll} \frac{Y_{i}}{Y_{i}^{eff}} & =\frac{{\left(\frac{s_{i}\beta_{Ki}}{{\bar{\beta }}_{K}}{\tilde{\gamma }}_{Ki}K\right)}^{\beta_{Ki}}{\left(\frac{s_{i}\beta_{Li}}{{\bar{\beta }}_{L}}{\tilde{\gamma }}_{Li}K\right)}^{\beta_{Li}}{\left(\frac{s_{i}\beta_{Mi}}{{\bar{\beta }}_{M}}{\tilde{\gamma }}_{Mi}M\right)}^{\beta_{Mi}}}{{\left(\frac{s_{i}\beta_{Ki}}{{\bar{\beta }}_{K}}K\right)}^{\beta_{Ki}}{\left(\frac{s_{i}\beta_{Li}}{{\bar{\beta }}_{L}}K\right)}^{\beta_{Li}}{\left(\frac{s_{i}\beta_{Mi}}{{\bar{\beta }}_{M}}M\right)}^{\beta_{Mi}}}\\ & ={\left({\tilde{\gamma }}_{Ki}\right)}^{\beta_{Ki}}{\left({\tilde{\gamma }}_{Li}\right)}^{\beta_{Li}}{\left({\tilde{\gamma }}_{Mi}\right)}^{\beta_{Mi}} \end{array}$$ (19)

The output gap of province *i* is:

$$E_{i}=1-\frac{Y_{i}}{Y_{i}{}^{eff}}=1-{\left({\tilde{\gamma }}_{Ki}\right)}^{\beta_{Ki}}{\left({\tilde{\gamma }}_{Li}\right)}^{\beta_{Li}}{\left({\tilde{\gamma }}_{Mi}\right)}^{\beta_{Mi}}$$ (20)

Combining Eqs (15) and (19), we can obtain:

$$\frac{Y}{Y^{eff}}={\prod_{i=1}^{N}\left(\frac{Y_{i}}{Y_{i}^{eff}}\right)}^{s_{i}}=\prod_{i=1}^{N}{\left[{\left({\tilde{\gamma }}_{Ki}\right)}^{\beta_{Ki}}{\left({\tilde{\gamma }}_{Li}\right)}^{\beta_{Li}}{\left({\tilde{\gamma }}_{Mi}\right)}^{\beta_{Mi}}\right]}^{s_{i}}$$ (21)

To calculate the country’s output gap *E*:

$$E=1-\frac{Y}{Y^{eff}}=1-\prod_{i=1}^{N}{\left[{\left({\tilde{\gamma }}_{Ki}\right)}^{\beta_{Ki}}{\left({\tilde{\gamma }}_{Li}\right)}^{\beta_{Li}}{\left({\tilde{\gamma }}_{Mi}\right)}^{\beta_{Mi}}\right]}^{s_{i}}$$ (22)

Eq (22) shows that the output gap of the whole country is determined by the relative distortion $\tilde{\gamma }$ of all factors and the share of production output of provinces *s*_*i*_ in the whole country.

### Output variation caused by relative factor distortion

This section will measure the output variation due to the variation in relative factor distortion among provinces, which has been mentioned before. In Eqs (13) and (14), part C depicts the output variation caused by the variation of the relative distortion coefficient of factors.

We assume that under competitive equilibrium only the distorted tax rate of one factor of a province changes (let’s assume the distorted tax rate of capital *K*_*i*_ of province *i* changes), for any province *j*(*j*≠*i*), the relative distortion coefficient of its capital *K*_*j*_ ${\tilde{\gamma }}_{Kj}=\frac{\gamma_{Kj}}{\sum_{n=1}^{N}\frac{s_{n}\beta_{Kn}}{{\bar{\beta }}_{K}}\gamma_{Kn}}$ changes to the same extent.

In reference to part C in Eq (14), we can get the output variation caused by the change in the distorted tax rate of capital *K*_*i*_ of province *i*:

$${ϒ}_{Ki}=\underset{A}{\underset{︸}{s_{i}\beta_{Ki}\cdot \Delta \;\text{ln}\;{\tilde{\gamma }}_{Ki}}}+\underset{B}{\underset{︸}{\sum_{j\neq i}s_{j}\beta_{Kj}\cdot \Delta \;\text{ln}\;{\tilde{\gamma }}_{Kj}}}$$ (23)

We specially extract Part A of province *i* from Eq (23), which is the output variation caused by the change of relative distortion coefficient ${\tilde{\gamma }}_{Ki}$ of province *i*; part B means that as *P*_*Ki*_ = (1 + *τ*_*Ki*_)*P*_*K*_ changes, the relative price of factor *K* of other provinces will change accordingly, which also brings changes to the capital allocation in other provinces. This is called relative price effect.

When the distorted tax rate of capital *K*_*i*_ of province *i* has a minor variation, the change in its absolute distortion coefficient Δ*γ*_*Ki*_ is minor. Meanwhile, the change in the relative distortion coefficient of other provinces is $\Delta \;\text{ln}\;{\tilde{\gamma }}_{Kj}\approx \frac{\Delta {\tilde{\gamma }}_{Kj}}{{\tilde{\gamma }}_{Kj}}\to 0$ and part B is negligible. Therefore, the output variation caused by the change in the distorted tax rate of province *i*’s capital *K*_*i*_ can be simplified into:

$${ϒ}_{Ki}\approx s_{i}\beta_{Ki}\cdot \Delta \;\text{ln}\;{\tilde{\gamma }}_{Ki}$$ (24)

### Factor misallocation measurement indicator

#### Factor allocation gap index

The methods of measuring the distortion of production factors can be divided into direct and indirect methods [24]. The direct method focuses on the causes of distortion and measures the proportion of subsidies or taxation of each unit of input; The indirect method, instead of focusing on the specific causes of distortion, analyzes the gap between the optimal and the actual allocations under profit maximization. In view of data availability, this paper aims to use an indirect method to calculate the factor allocation gap index of each province:

$$\left\{\begin{array}{l} DIS_{Ki}=\frac{MP_{Ki}}{MP_{Ki}^{eff}}=\frac{MP_{Ki}}{P_{K}}=\frac{\beta_{Ki}P_{i}Y_{i}/K_{i}}{P_{K}}\\ DIS_{Li}=\frac{MP_{L}}{MP_{Li}^{eff}}=\frac{MP_{Li}}{P_{L}}=\frac{\beta_{Li}P_{i}Y_{i}/L_{i}}{P_{L}}\\ DIS_{Mi}=\frac{MP_{M}}{MP_{Mi}^{eff}}=\frac{MP_{Mi}}{P_{M}}=\frac{\beta_{Mi}P_{i}Y_{i}/M_{i}}{P_{M}} \end{array}\right.$$ (25)

where *MP* is the marginal output of a factor; *MP*^*eff*^ is the marginal output of a factor without distortion which is equal to the factor price under profit maximization. Under competitive equilibrium, the index of factor allocation gap should be 1. When the index is greater than 1, the marginal output of a factor exceeds the factor price, and the factor input is less than the optimal input under effective output, in other words, the factor allocation is inefficient. In contrast, when the index is less than 1, the marginal output of a factor is less than the factor price, leading to surplus factor allocation. The comprehensive allocation gap index of all factors of province *i* is:

$${\bar{DIS}}_{i}=\beta_{Ki}\cdot DIS_{Ki}+\beta_{Li}\cdot DIS_{Li}+\beta_{Mi}\cdot DIS_{Mi}$$ (26)

The comprehensive allocation gap index can summarize the allocation of all factors in this province.

#### Land misallocation index

Small per capita land use is a fundamental characteristic of China, especially in the eastern coastal areas. The most frequently-used indicator to measure the efficiency of land use is *APM* which can work out the production output per unit land area.


$$APM=\frac{\text{real}\;\text{GDP}}{\text{total}\;\text{land}\;\text{area}}$$ (27)

Following Duranton and Turner [28], we define the misallocation of land across provinces in year t as follows:

$$Mis_{t}=N\cdot \text{cov}(\frac{M_{it}}{M_{t}},TFP_{it})$$ (28)

where *N* is the number of provinces; *M*_*it*_/*M*_*t*_ is province *i*’s share of built-up land area in year t; *TFP*_*it*_ is the total factor productivity in province *i* in year t. This indicator is very intuitive: if the provinces with higher TFP have larger shares of land, *Mis*_*t*_ would be smaller and there is less misallocation. Otherwise, if the provinces with higher TFP have smaller shares of land, *Mis*_*t*_ would be larger and there is worse allocation.

## Empirical analysis

In this chapter, the analytical framework mentioned above is applied to measure regional resource allocation efficiency and output loss due to resource misallocation in China. The data from China’s 31 provinces and their sub-prefectural cities were collected for the estimation of the production function and TFP of each province and further calculation of the relative distortion coefficients of the factor price.

### Estimation of the production function

#### Data source

To measure the allocation of production factors in China, data at the provincial and municipal levels are essential. First, estimating China’s production function requires provincial-level data, including the output value *Y*_*i*_, capital input *K*_*i*_, labor input *L*_*i*_, and land use *M*_*i*_ in each province. Second, estimating the production function of each province requires municipal-level data on the above variables. This section highlights the key procedures and sources of data. All data used in this paper are from the National Bureau of Statistics of China (NBS) and the China Statistical Yearbook from 2004 to 2020.

Output. The real GDP of each province is used to represent output. The data on the nominal GDP of each province from 2000 to 2020 is obtained from the National Bureau of Statistics (NBS). Taking into account the impact of inflation, the GDP index at constant prices of each province is adopted to calculate the real GDP of each province based on the year 2000eriod. The real GDP of all cities in each province is calculated in the same way.

Capital. The capital stock of each province is adopted to represent capital. Since the data cannot be obtained directly, based on the method proposed by Goldsmith [29], this paper utilizes the perpetual inventory system to estimate the capital stock of each province at the end of the year from 2000 to 2020. The equation is as follows:

$$K_{it}=K_{it-1}(1-\delta_{it})+I_{it}$$ (29)

where *K*_*it*_ is the physical capital stock of the province *i* in the year *t*, which is equal to the depreciated capital stock of the previous year plus the new investment *I*_*it*_ in the current year. The depreciation rate *δ* in each province is 9.6%. The nominal Gross Fixed Capital Formation (GFCF) of each province is deflated using the investment implicit price deflator to get the actual GFCF as a measurement of *I*_*it*_. The data on the annual nominal GFCF and the investment implicit price deflator in each province are from the China Statistical Yearbook. More details on the specific calculation process can be referred to Goldsmith [29]. With the same approach, the real capital stock *K*_*it*_ and the annual increase in real investment *I*_*it*_ of all cities in each province are calculated.

Labor. The number of employed is adopted to represent labor. Since a large number of employees in China are temporary workers or are engaged in multiple part-time jobs, the number of employed is used to represent labor input *L*_*i*_ rather than the summation of staff in companies. The data on the number of employed in each city and province from 2000 to 2020 is extracted from the reports released by the Ministry of Human Resources and Social Security of the People’s Republic of China.

Land. This paper adds land as a factor of production, making it distinguishable from other studies. As China has a huge population and a relatively smaller land area per capita, the land area and the cost of land use constitute major concerns for the manufacturers, especially for those in the eastern coastal regions. Therefore, land is also a factor of production that shall not be neglected in China. Considering that there is still a large amount of undeveloped land in each province, it is not appropriate to directly use the administrative area of each province to represent land. Therefore, the built-up area of each region is used to represent land *M*_*i*_. The built-up area of the province *i* is obtained by summing the area of all its subordinate cities. The built-up area refers to the actual constructed area for production with non-agricultural activities, which includes the concentrated urban area and the urban construction sites dispersed in the suburbs with well-established municipal utilities. Therefore, it can be assumed that nearly all production activities take place in the built-up area. Since the Ministry of Housing and Urban-Rural Development of the People’s Republic of China did not release such data until 2004, only the data from 2004 to 2020 are included.

Table 1 presents the statistical description of the data collected based on the above description of data sources. These data were rooted in 275 cities in 31 provinces of China from 2004 to 2020. Besides, Table 2 more clearly shows the variation trend of these variables over time in the Chinese mainland. In addition, it is worth noting that in both Tables 1 and 2, the values of other variables are all adjusted by the GDP deflator except for the two variables: labor input and built-up area.

**Table 1:** The statistical description of variables.

Variables	Unit	Obs	Mean	Std	Min	Median	Max
GDP Deflator	(unitless number)	4675	1.44	0.25	0.96	1.43	2.17
Real GDP	billion CNY	4,675	1,190.6	1,991.4	6.6	601.8	27,129.7
Capital Stock	billion CNY	4,653	4,530.5	6,252.0	114.8	2,476.6	78,441.6
Labor Input	million people	4,567	67.1	115.5	0.2	30.0	1,283.4
Built-up Area	square kilometer	4,487	129.2	211.1	1.0	70.0	2,915.6
Annual Investment	billion CNY	4,653	892.1	1,114.7	19.7	523.6	12,724.7
Annual Salary	CNY	4,567	40,790.7	21,391.2	11,855.0	37,911.0	131,700.0
Land Price	CNY per square meter	4,487	1,278.4	2,803.0	36.9	618.4	29,771.7

Table 1 shows city-level data from the prefecture-level municipal statistics Bureau of 275 cities in 31 provinces from 2004 to 2020. The variable Annual Salary represents the average salary of urban workers in each city. The variable Land Price equals each city government’s yearly land transfer fee income divided by the annual land transfer area. The following empirical analysis will also explain these two variables in detail.

**Table 2:** The values of variables of the Chinese mainland.

Variables	Unit	2004	2008	2012	2016	2020
GDP Deflator	(unitless number)	1.00	1.25	1.48	1.55	1.70
Real GDP	billion CNY	143,498.7	225,699.1	322,949.4	427,279.8	528,489.2
Capital Stock	billion CNY	29,649.9	55,379.1	103,243.8	163,858.5	232,660.9
Labor Input	million people	742.6	755.6	762.5	762.5	750.6
Built-up Area	square kilometer	30,406.2	36,295.3	45,565.8	54,331.5	60,721.3
Annual Investment	billion CNY	6,409.2	12,476.3	22,421.7	29,745.6	49,503.1
Average Annual Salary	CNY	5,660.9	7,938.7	11,165.0	15,379.5	18,883.9
Average Land Price	CNY per square meter	352.2	491.0	570.9	1,113.4	2,251.8

The data in Table 2 is collected from the National Bureau of Statistics of China. The variable Annual Salary represents the average salary of urban workers in China. The variable Average Land Price equals the total annual national land transfer revenue divided by the total land transfer area. The following empirical analysis will also explain these two variables in detail.

#### Estimation method

Due to the simultaneity bias in OLS, most literature uses the method proposed by Olley and Pakes [30] to estimate the production function. Based on the Olley-Pakes method, the production function is estimated as follows:

$$\text{ln}\;Y_{it}=\alpha +\beta_{Ki}\;\text{ln}\;K_{it}+\beta_{Li}\;\text{ln}\;L_{it}+\beta_{Mi}\;\text{ln}\;M_{it}+\omega_{it}+\varepsilon_{it}$$ (30)

where ln*TFP*_*it*_ is resolved into two parts: the constant term *α* and the random component *ω*_*it*_ which is the unobservable productivity or technical efficiency. *ε*_*it*_ is an idiosyncratic shock distributed as white noise. *K*_*it*_ is taken as the state variable, *L*_*it*_ and *M*_*it*_ as free variables, the investment amount of the province in the current year *I*_*it*_ as the proxy variable.

Following the Olley-Pakes method, the random component *ω*_*it*_, which is a part of ln*TFP*_*it*_, evolves according to a first-order Markov process:

$$\omega_{it}=Ε\left(\omega_{it}|\omega_{it-1}\right)+\xi_{it}=f(\omega_{it-1})+\xi_{it}$$ (31)

where *ω*_*it*−1_ can also be considered as information set at time t-1. Moreover, *ω*_*it*_ is the function of proxy variable *I*_*it*_ and state variable *K*_*it*_. *ξ*_*it*_ represents productivity shock, which is independent of *ω*_*it*_ and state variable *K*_*it*_.

#### Estimation results

With reference to the Olley-Pakes method, the national production function is estimated based on the municipal-level data from 2004 to 2020. Each city is divided by its location into east China, middle China, and west China, and its production function is also estimated. The estimated results are shown in Table 3.

**Table 3:** Estimates of the national and regional production function.

Coefficient	China	East	Middle	West
Capital	0.551***	0.529***	0.599***	0.532***
	(16.7637)	(7.1569)	(14.2910)	(9.8792)
Labor	0.203***	0.180***	0.104***	0.240***
	(9.8249)	(4.6777)	(4.4202)	(4.7307)
Land	0.255***	0.229***	0.256***	0.190***
	(10.6002)	(6.5385)	(5.1065)	(4.4661)
Observations	4,748	1,700	1,698	1,350
Number of Groups	275	98	96	81

z-statistics in parentheses.

*** p<0.01,

** p<0.05,

* p<0.1.

According to the regression results, it can be found that the sum of the coefficients of capital, labor, and land is approximately equal to 1. The F-test is conducted, failing to reject the null hypothesis ${\hat{\beta }}_{K}+{\hat{\beta }}_{L}+{\hat{\beta }}_{M}=1$, which indicates that production in China is characterized by constant returns to scale. It is worth noting that the output elasticity ${\hat{\beta }}_{K}$ of capital, both nationally and regionally, is the largest, accounting for more than half, which reveals that production in China is highly dependent on capital inputs. The elasticity of land ${\hat{\beta }}_{M}$ is basically equivalent to that of labor ${\hat{\beta }}_{L}$, but the elasticity of labor ${\hat{\beta }}_{L}$ is slightly smaller. In addition, the capital and land contribute more to production output in east and middle China than that in west China, while the labor contributes more to output in west China than that in the east and middle China. In general, there are no major differences in the elasticity of the various factors in east, middle, and west China, which generally validates the regression results in this paper.

#### TFP in each province and region

According to the Eq (30), the residual of the regression equation is the logarithmic TFP, which is:

$$\text{ln}\;TFP_{it}=\text{ln}\;Y_{it}-\text{ln}\;{\hat{Y}}_{it}=\text{ln}\;Y_{it}-{\hat{\beta }}_{Ki}\;\text{ln}\;K_{it}-{\hat{\beta }}_{Li}\;\text{ln}\;L_{it}-{\hat{\beta }}_{Mi}\;\text{ln}\;M_{it}$$ (32)

Therefore, the estimated value of the output elasticity of each factor calculated in the previous chapter can be used to calculate the logarithmic TFP ln*TFP*_*it*_. The TFP of each province is calculated separately, and Table 4 shows the calculation results in some years. In addition, in order to show the differences in production in different regions, 31 provinces are divided into three main regions, namely East China, Middle China, and west China, and the TFPs of these three regions are calculated with weighting for the output value of each province. The calculation results are shown in Table 5.

**Table 4:** Productivity of 31 provinces (ln TFP).

Province	2004	2008	2012	2016	2020	Average	Ranking
Shanghai	1.1619	1.3019	1.4025	1.5175	1.4401	1.3648	1
Fujian	1.0691	1.0751	1.0611	1.0651	1.0544	1.0650	2
Beijing	0.7499	0.8782	0.9939	0.9854	1.0354	0.9286	3
Guangdong	0.8601	0.9467	0.9455	0.9224	0.8646	0.9079	4
Jiangsu	0.7918	0.8720	0.9023	0.9454	0.9541	0.8931	5
Zhejiang	0.8127	0.8601	0.8981	0.9366	0.9354	0.8886	6
Hainan	0.8330	0.9358	0.9210	0.8699	0.8051	0.8730	7
Hunan	0.7348	0.8339	0.8674	0.9209	0.9220	0.8558	8
Chongqing	0.7441	0.7619	0.8557	0.9101	0.9162	0.8376	9
Jiangxi	0.7132	0.7510	0.7931	0.8193	0.7971	0.7747	10
Anhui	0.6379	0.7315	0.7716	0.7936	0.7949	0.7459	11
Tianjin	0.8203	0.8218	0.8152	0.6813	0.5782	0.7433	12
Sichuan	0.5607	0.7262	0.8118	0.7653	0.7562	0.7240	13
Hubei	0.5902	0.7048	0.7613	0.7548	0.5915	0.6805	14
Inner Mongolia	0.6961	0.6938	0.6451	0.6607	0.6816	0.6755	15
Liaoning	0.7188	0.6807	0.6440	0.6106	0.6698	0.6648	16
Gansu	0.6206	0.6328	0.7004	0.6494	0.7061	0.6618	17
Yunnan	0.7696	0.7173	0.6605	0.5733	0.5437	0.6529	18
Shaanxi	0.6249	0.6887	0.6806	0.6453	0.5806	0.6440	19
Heilongjiang	0.5580	0.6523	0.6607	0.6465	0.6393	0.6313	20
Henan	0.6690	0.6575	0.6113	0.6017	0.5824	0.6244	21
Shandong	0.6391	0.6051	0.5948	0.6124	0.5963	0.6095	22
Qinghai	0.5673	0.7238	0.7638	0.4756	0.3431	0.5747	23
Shanxi	0.7143	0.6627	0.5123	0.4413	0.4945	0.5650	24

**Table 5:** Productivity of China and regions (ln TFP).

Region	2004	2008	2012	2016	2020	Average
China	0.7421	0.7891	0.7970	0.7983	0.7800	0.7813
East	0.8154	0.8642	0.8805	0.8930	0.8791	0.8664
Middle	0.6451	0.6846	0.6876	0.6977	0.6710	0.6772
West	0.6228	0.6743	0.6811	0.6469	0.6272	0.6505

It is intuitive from Table 4 that there are significant differences in TFP in different provinces. The TFP in the selected years is averaged and then ranked. It is found that the top-ranking provinces are basically in east China, the mid-ranking provinces are in middle China, and the provinces ranking behind are all in west China. Along with time, from 2004 to 2020, most provinces located in east and middle China witnessed an increase in the TFP, but basically all provinces in west China experienced a decrease in the TFP to varying degrees in the last decade. It is noteworthy that, this paper divides the 31 provinces of Chinese mainland into east, middle, and west China according to their geographical locations. Specifically, east China includes Beijing, Tianjin, Hebei, Liaoning, Shanghai, Jiangsu, Zhejiang, Fujian, Guangdong, Hainan, and Shandong; middle China includes Shanxi, Jilin, Heilongjiang, Anhui, Jiangxi, Henan, Hubei, and Hunan; west China includes Inner Mongolia, Guangxi, Chongqing, Sichuan, Guizhou, Yunnan, Tibet, Shaanxi, Gansu, Qinghai, Ningxia, and Xinjiang.

**Fig 1:**
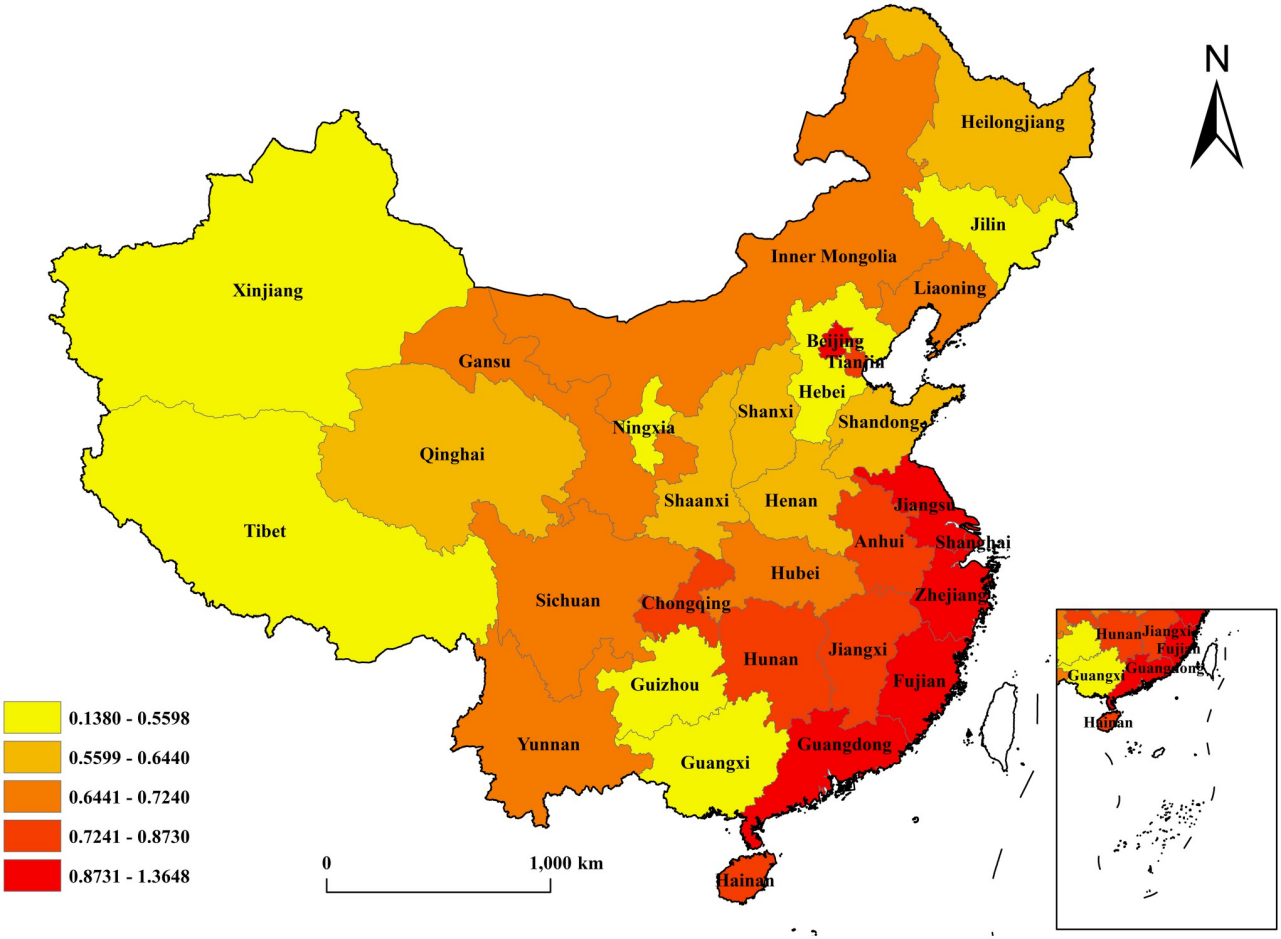
Logarithm of TFP of each province. The basemap of China was downloaded from the official website of the National Geomatics Center of China (NGCC) at http://www.ngcc.cn/ngcc/. The data used was calculated by the author.

To further demonstrate the differences of TFP in different regions, the values of TFP in different regions are shown in Table 5. It can be easily seen that the TFP of east China is significantly larger than those of middle China and west China, and the TFPs of middle China and west China are both below the national level. The calculation result is also in line with the notion that economic development is more developed in east China, and is relatively backward in middle and west China. Based on the data on average TFP in Table 4, a map (Fig 1) is drawn to more intuitively show the development differences between east and west China.

### Relative distortion coefficient of factor price

After the estimation of the elasticity of the three factors, the relative distortion coefficients of these factor prices in each province can be calculated. To make the data more intuitively displayed, the relative distortion coefficients of factor prices in different regions are calculated with the output value of each province as weights. Table 6 indicates the distortions in the allocation of capital, labor, and land from 2004 to 2020.

**Table 6:** Relative distortion coefficient.

		2004	2008	2012	2016	2020
Relative Distortion Coefficient of Capital ${\hat{\gamma }}_{K}$	East	1.0188	0.9866	0.9568	0.9262	0.9156
	Middle	1.0188	0.9866	0.9568	0.9262	0.9156
	West	1.1365	1.1624	1.1835	1.2271	1.2313
Relative Distortion Coefficient of Labor ${\hat{\gamma }}_{L}$	East	0.6288	0.6612	0.6984	0.7248	0.7564
	Middle	0.6288	0.6612	0.6984	0.7248	0.7564
	West	1.1907	1.1528	1.0423	1.0201	1.0027
Relative Distortion Coefficient of Land ${\hat{\gamma }}_{M}$	East	0.8701	0.8910	0.8933	0.8954	0.8819
	Middle	0.8701	0.8910	0.8933	0.8954	0.8819
	West	1.3909	1.3539	1.3710	1.4210	1.4328

According to the conclusions drawn from the previous analysis, a relative distortion coefficient greater than 1 indicates that the factor price in the region is relatively low compared with the overall level, and that the factor is over-allocated in this region. In contrast, a relative distortion coefficient less than 1 indicates that the factor price in the region is relatively high compared with the overall level, and that the factor is under-allocated in this region. It can be seen from the results in Table 6 that the capital, labor, and land are all under-allocated in east and middle China, but over-allocated in west China. Obviously, the costs of factors in east and middle China are higher than those in west China, which seems to violate the Law of One Price. What hinders the flow of resources between regions? In fact, the GDP of east and middle China accounts for more than 80% of China’s total GDP, and the vast majority of production activities take place in these regions. For the sole goal of increasing GDP, the Chinese government is supposed to allow more resources to flow from the west to the eastern and middle regions. However, to narrow the gap between the rich and the poor and maintain coordinated development among regions, the Chinese government implemented the Western Development Program in the 1990s. This policy caused a large flow of production factors and resources from the east and the middle to the west. The government offers subsidies and tax incentives to manufacturing enterprises in western China to encourage them to expand their production scale and ultimately increase GDP. This explains why the western region has access to cheaper capital, labor, and land for production than the eastern and middle regions. The central government’s policy hinders the flow of factors of production between regions.

### Estimation of output gap

After the calculation of relative distortion coefficients for each province, the output gap between actual output and potential output can be estimated. Based on the results deduced in the previous chapter, the value of the national output gap can be calculated:

$$E=1-\frac{Y}{Y^{eff}}=1-\prod_{i=1}^{N}{\left[{\left({\tilde{\gamma }}_{Ki}\right)}^{\beta_{Ki}}{\left({\tilde{\gamma }}_{Li}\right)}^{\beta_{Li}}{\left({\tilde{\gamma }}_{Mi}\right)}^{\beta_{Mi}}\right]}^{s_{i}}$$ (33)

**Fig 2:**
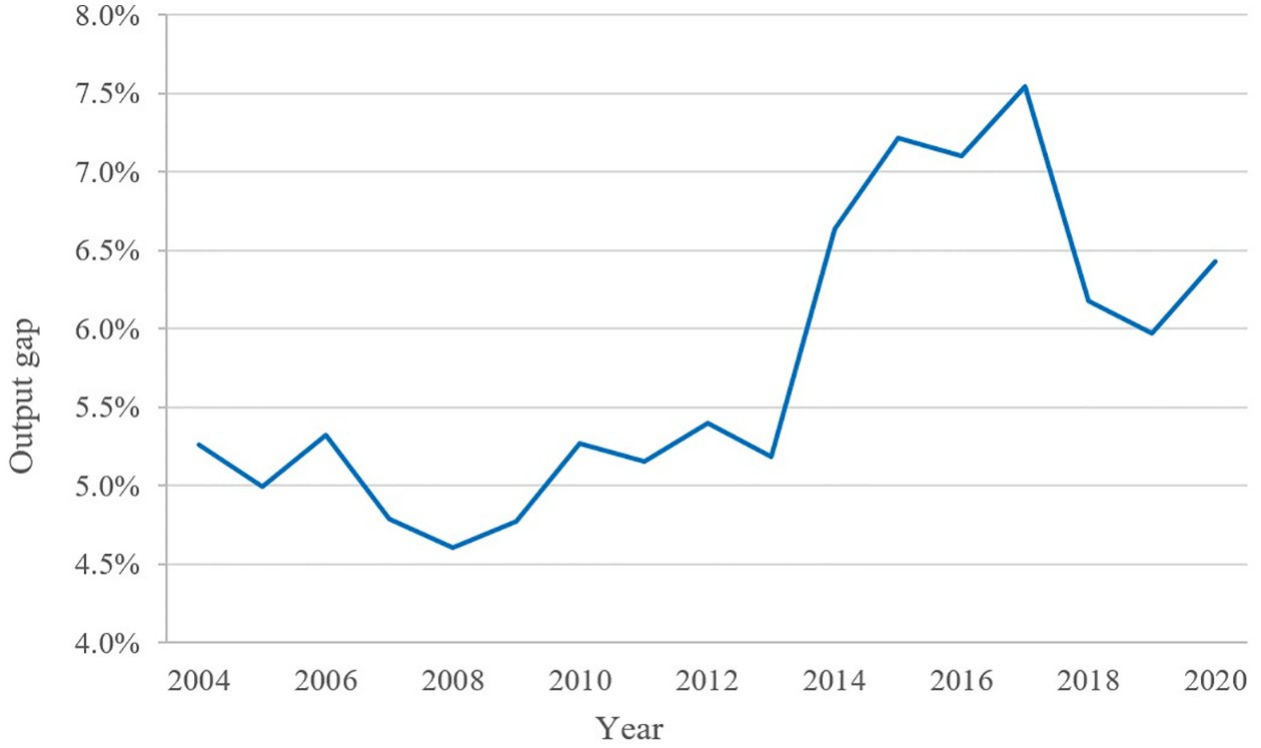
Output gap. The data used is the author’s calculations using Stata, and the graph was drawn in Excel.

To better reflect the changes in the output gap from 2004 to 2020, the values of the output gap in each year are plotted as a line graph, shown in Fig 2.

From Fig 2, it can be seen that from 2004 to 2012, the output gap was basically maintained at 5% with less variance. In 2014, the output gap increased considerably and peaked in 2017 (7.5450%). From 2012 to 2020, the output gap increased by a notch, nearly 7%. Meanwhile, China’s economic growth slowed down. If factor allocation can be improved, GDP can be increased by 6.5%, which is a very impressive growth. It indicates that to boost the economic growth of a country, in addition to improving the TFP and increasing resource input, relieving factor price distortion between different regions is also a good approach.

### Analysis of the reasons for economic growth

This section analyzes the factors driving China’s economic growth. According to Syrquin’s model [27] for economic growth mentioned in the previous chapter, the equation is as follows (34). Following this method, the contribution of these four factors to China’s economic growth from 2005 to 2020 is calculated.


$$\begin{array}{ll} \Delta \;\text{ln}\;Y_{t} & =\text{ln}\;Y_{t+1}-\text{ln}\;Y_{t}=\sum_{i=1}^{N}s_{it}\Delta \;\text{ln}\;Y_{it}\\ & =\underset{\text{A}}{\underset{︸}{\sum_{i=1}^{N}s_{it}\Delta \;\text{ln}\;TFP_{i}}}+\underset{\text{B}}{\underset{︸}{\sum_{i=1}^{N}s_{it}\;\text{ln}[\left(\frac{s_{it+1}}{s_{it}}\right)/\left(\frac{{\bar{\beta }}_{Kt+1}^{\beta_{Ki}}{\bar{\beta }}_{Lt+1}^{\beta_{Li}}{\bar{\beta }}_{Mt+1}^{\beta_{Mi}}}{{\bar{\beta }}_{Kt}^{\beta_{Ki}}{\bar{\beta }}_{Lt}^{\beta_{Li}}{\bar{\beta }}_{Mt}^{\beta_{Mi}}}\right)]}}\\ & +\underset{\text{C}}{\underset{︸}{\sum_{i=1}^{N}s_{it}\left[\beta_{Ki}\Delta \;\text{ln}\;{\tilde{\gamma }}_{Ki}+\beta_{Li}\Delta \;\text{ln}\;{\tilde{\gamma }}_{Li}+\beta_{Mi}\Delta \;\text{ln}\;{\tilde{\gamma }}_{Mi}\right]}}\\ & +\underset{\text{D}}{\underset{︸}{\sum_{i=1}^{N}s_{it}\left[\beta_{Ki}\Delta \;\text{ln}\;K+\beta_{Li}\Delta \;\text{ln}\;L+\beta_{Mi}\Delta \;\text{ln}\;M\right]}} \end{array}$$ (34)

The calculation results are shown in Table 7. The left side of the equation represents the economic growth, which is driven by: the growth of TFP (part A), accounting for 32.83%, and the increase of factor input (part D), accounting for 65.56%. The change of factor elasticity (part B) and the improvement of factor price distortion (part C) make a negligible contribution to the economic growth, accounting for only 1.61%. This shows that as the output gap caused by factor price distortion has not been reduced, the misallocation of production factors and resources has not been significantly improved. China still has the potential to increase GDP by correcting distortions in resource allocation. On one hand, it is gratifying to note that the growth in TFP has made an increasing contribution to China’s economic growth. On the other hand, the increase in factor inputs plays a less important role. This also demonstrates that from the perspective of production activities, China’s economic growth has gradually transformed from an extensive growth model driven by increasing resource input to an intensive growth model driven by technological progress.

**Table 7:** Analysis of the economic growth.

Year	2007	2009	2011	2013	2015	2017	2019	Average
Left Side of Equation	3.968536	3.319641	3.520203	2.906799	2.336182	2.154236	1.907104	2.813266
Part A	0.791135	0.787959	0.801292	0.799644	0.802863	0.815991	0.842635	0.797723
*Proportion of A*	*19*.*94%*	*23*.*74%*	*22*.*76%*	*27*.*51%*	*34*.*37%*	*37*.*88%*	*44*.*18%*	*32*.*83%*
Part B	-0.00457	0.04128	0.09769	0.04823	0.0318	0.01819	0.00859	0.037649
*Proportion of B*	*-0*.*12%*	*1*.*24%*	*2*.*78%*	*1*.*66%*	*1*.*36%*	*0*.*84%*	*0*.*45%*	*1*.*56%*
Part C	0.024805	-0.01154	-0.01243	0.014196	-0.00375	0.008721	0.011926	0.002056
*Proportion of C*	*0*.*63%*	*-0*.*35%*	*-0*.*35%*	*0*.*49%*	*-0*.*16%*	*0*.*40%*	*0*.*63%*	*0*.*05%*
Part D	3.157166	2.501943	2.633647	2.044729	1.505266	1.311334	1.043953	1.975838
*Proportion of D*	*79*.*55%*	*75*.*37%*	*74*.*82%*	*70*.*34%*	*64*.*43%*	*60*.*87%*	*54*.*74%*	*65*.*56%*

**Fig 3:**
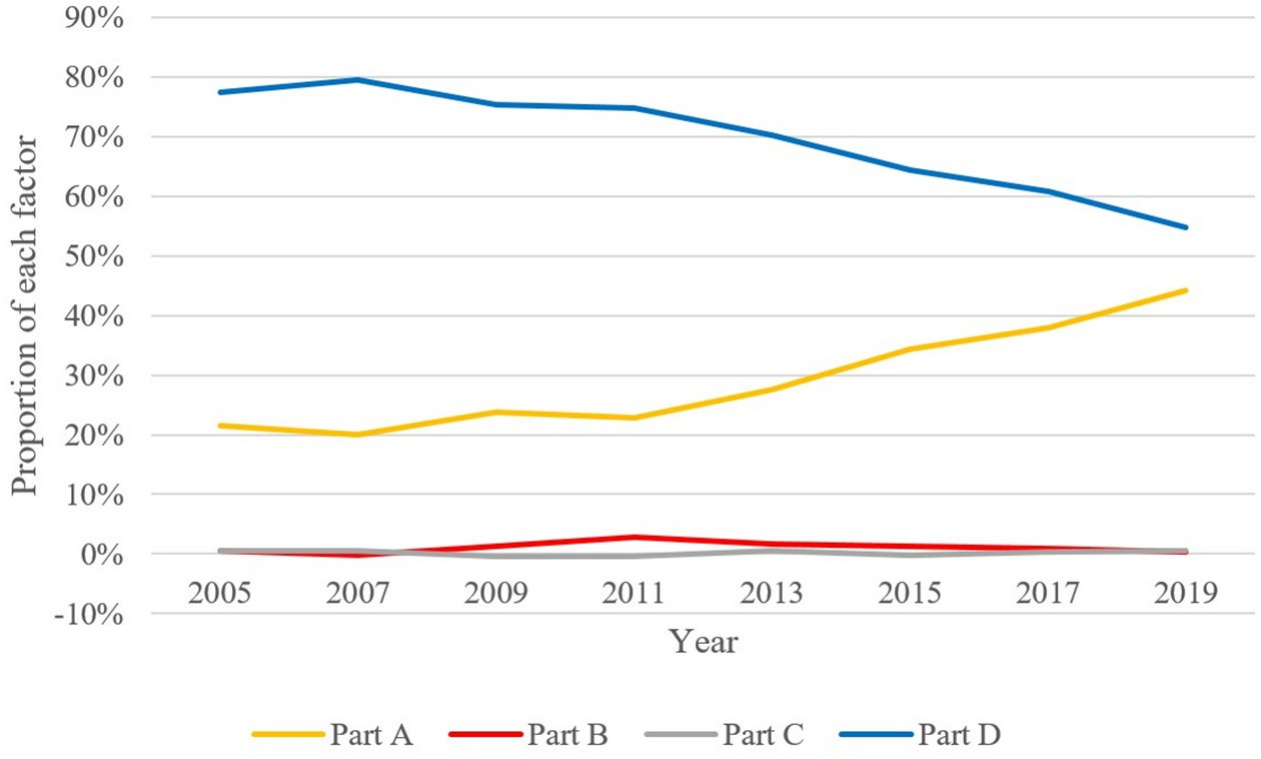
Analysis of the driving factors of economic growth. The data used is the author’s calculations using Stata, and the graph was drawn in Excel.

The proportions of each part in Table 7 are graphed in the line chart below. Fig 3 visualizes more intuitively the contribution of the four factors driving China’s economic growth.

### Contribution of production factors to economic growth

**Fig 4:**
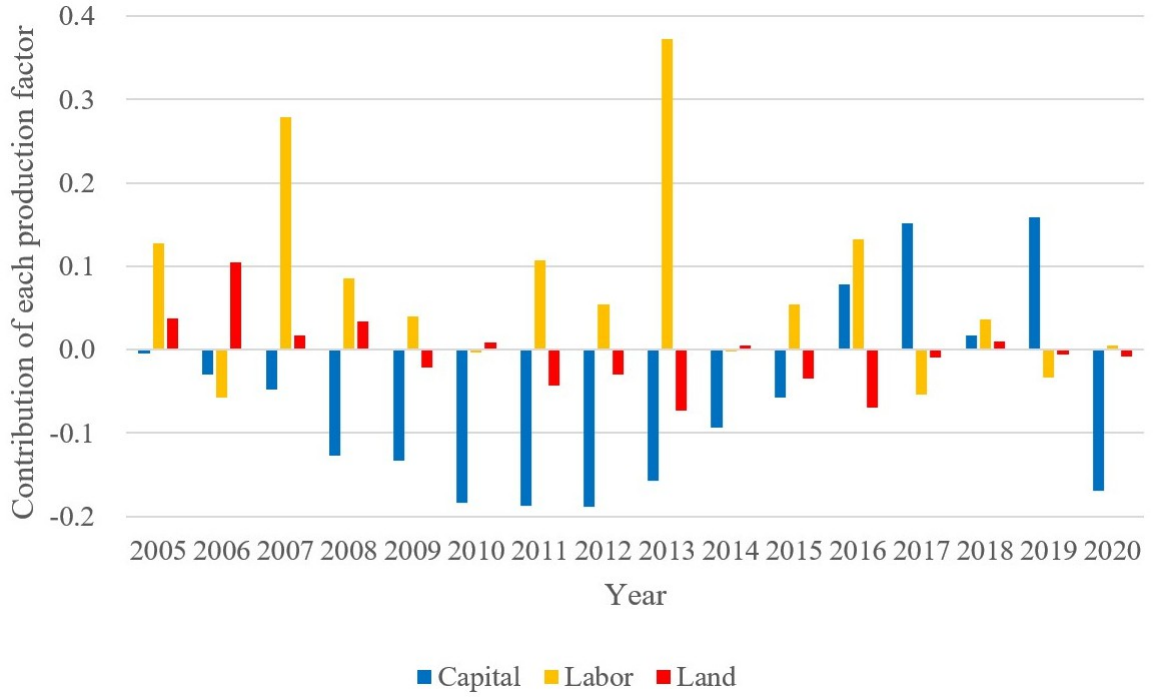
Contribution of changes in relative distortion coefficients to economic growth. The data used is the author’s calculations using Stata, and the graph was drawn in Excel.

According to the calculation results in the previous section, part C in Eq (34) is subdivided, that is, the specific contribution value of capital, labor, and land to economic growth is further calculated. The calculation results are plotted in Fig 4. It can be found that the contribution of labor is greater than zero most of the time and is the largest among the three production factors. The contribution of land fluctuates slightly around zero. The contribution of capital is often negative, which is the smallest among the three production factors. In general, the change in labor price distortion promotes economic growth, that in land price distortion contributes little to economic growth, and that of capital price distortion hinders economic growth.

### Configuration gap of measurement elements

The approach proposed by Restuccia and Rogerson [24] is adopted to measure the distortion of factor allocation. The ratio between the actual marginal output of factors and the marginal output of competitive equilibrium is calculated, that is, the allocation gap coefficient of the production factor. The 5-year local government bond rates represent the cost of capital and the average salaries of urban workers as the price of labor. In China, manufacturers generally purchase land from the government and pay in a lump sum, with a 30-year term of use of the land. Therefore, the transfer price of state-owned land in the current year is averagely amortized over 30 years at an annual interest rate of 10%, and the annual amortization amount is taken as the cost of land. Since only the data on factor prices from 2004 to 2017 is collected, the calculation results in the Table 8 are the average values of the annual allocation gap coefficient of this period. In order to have a more comprehensive picture of the situation of factor allocation in different regions, the weighted mean of the gap coefficient of factor allocation is calculated based on the output elasticity of each factor. The weighted mean can better reflect the overall situation of factor allocation in a certain region.

**Table 8:** Gap index of the factor allocation.

	China	East	Middle	West
K	1.1743	1.1353	1.089	1.3859
L	0.5965	0.6761	0.3169	0.7093
M	2.9120	2.3698	3.7702	3.4
Weighted Mean	1.5107	1.265	1.6504	1.5535

According to the previous explanation of the coefficient, when the coefficient is greater than 1, the marginal output of the factor is greater than the price of the factor, that is, the factor’s contribution to the output reis greater than its cost. In this regard, it is wise for manufacturers to increase the input of this factor. On the contrary, when the coefficient is less than 1, the factor’s contribution to output is less than its cost, and manufacturers should reduce the input of this factor. According to the calculation results in Table 8, it is found that the gap coefficient of capital allocation is slightly higher than 1, which means that the general allocation of capital is reasonable. However, the gap coefficient of labor allocation is only 0.6, indicating that the salary paid by the enterprise to the employee is far greater than the contribution of the employee to the output value. Do enterprises need massive layoffs? This is a result caused by the misalignment of objects in statistics, in that many workers in China are temporary workers who are not included in the official staff of enterprises. Their salaries are far lower than those of the formal workers. Although they are included in the statistics of the number of employees, their salaries are not included. For tax and reputation reasons, enterprises would not report the salaries of temporary workers to the National Bureau of Statistics (NBS), but only release the salaries of formal workers to show off what they pay for their employees. In other words, the income of informal employees was not counted by the statistics bureau, but only the income of formal employees. To some extent, this explains why the gap coefficient of labor allocation is less than 1. Besides, the gap coefficient of land allocation in China is approximately 3, indicating that the contribution of land to production output value is much greater than the land cost. In China, the price of land purchased by enterprises from the government is less than half of the average price of residential houses., in that the government sells the land use right at a low price to promote enterprises to carry out production and construction. During the term of land use, the value created by the use of land by enterprises is far greater than the land cost [31,32]. The government should reduce its intervention in the land market to make it more open for competition, so as to improve the efficiency of land use [33,34]. In general, the weighted mean of the gap coefficient of factor allocation in all regions is greater than 1, that is, the marginal output of factors is greater than their cost, indicating that the cost of production factors in China is generally low.

### Misallocation of land across regions

**Fig 5:**
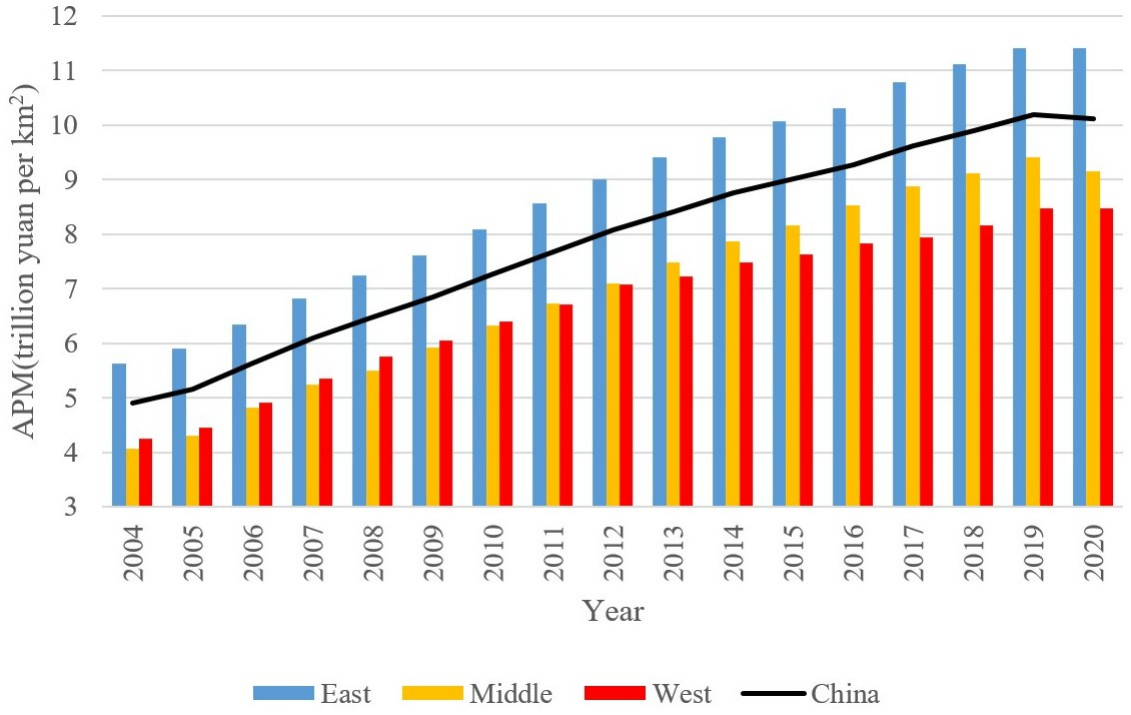
APM in differentregions. The data used is the author’s calculations using Stata, and the graph was drawn in Excel.

By dividing the output value of each region by its built-up area, the average production per square kilometer of land (APM) of the region is calculated. The calculation results are shown in Fig 5. It is found that the APM in east China is significantly higher than that in middle and west China. The APM in middle China is nearly identical to that of west China, but the gap has been gradually increasing over the past five years. Along with time, the growth rate of APM in these three regions is consistent. From 2004 to 2020, the average annual growth rate of the national APM was 4.65%, and the average growth rate of the national GDP was 8.5%. In addition, it is found that the growth of APM and GDP are highly positively correlated, and that the correlation coefficient between the growth rates of the two is as high as 0.95.

**Fig 6:**
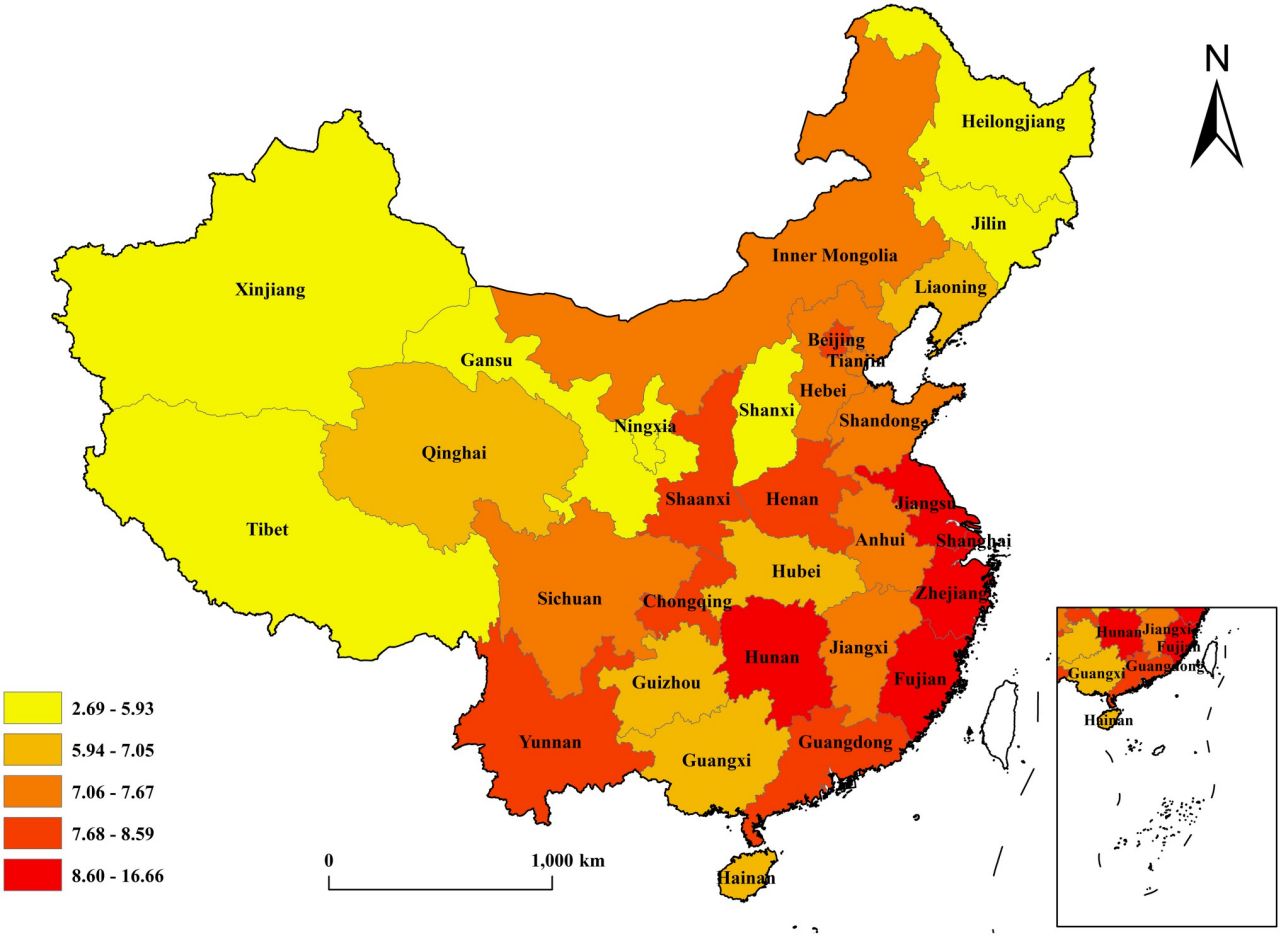
APM in different provinces. The basemap of China was downloaded from the official website of the National Geomatics Center of China (NGCC) at http://www.ngcc.cn/ngcc/. The data used was calculated by the author.

To better understand the APM in each province in China, the average values of APM in each province from 2004 to 2020 are calculated and plotted in Fig 6. From the map below, it can be found that the average value of APM is progressively decreasing from east China to middle China and then to west China.

Based on the method of Duranton and Turner [28], the misallocation coefficient of land in China is calculated. The calculation results are shown in Table 9. It is found that this coefficient increases year by year, which indicates that the distortion of land allocation has been improved. Provinces with high TFP are granted more land quotas, as the government allows these provinces to increase land input for production. Furthermore, the misallocation coefficients of labor and capital are calculated using the equation. The misallocation coefficient of labor is growing the fastest among the three factors, indicating that the allocation of labor has also been improved, and regions with high productivity attract more workers. The misallocation coefficient of capital is the largest among the three factors, indicating that the allocation of capital is optimal. It is worth noting that the misallocation coefficients of all factors have tended to move towards convergence since 2013, indicating an important fact that the share of all factors owned by a province tends to be equal, that is, the proportion of factor input of a province in the national total is close to those of other factors. On the whole, factors of production gradually flowed to regions with high TFP.

**Table 9:** Misallocation indices of factors.

Year	2004	2008	2012	2016	2020	Mean
Land	1.197434	1.593285	1.595347	2.322799	2.624017	2.008003
Capital	3.118417	2.866403	1.854367	2.491207	2.794926	2.701007
Labor	0.070708	0.501019	0.798008	2.15038	2.263109	1.267207

Some attentive readers may find that this paper seems to have reached a contradictory conclusion that while the misallocation coefficients of factors are getting smaller, the output gap is getting larger. But actually this is not contradictory. First of all, the two indicators are used to describe different conditions. The misallocation coefficients of factors measure the dispersion of factor shares and TFP. The smaller the factor misallocation coefficient, the more resources can be obtained for production in regions with high TFP. The output gap measures the loss of efficiency caused by the factor price distortion. The increase in output gap means that the gap between the costs of resources in different regions becomes larger, resulting in a greater loss of the output value. To sum up, the misallocation coefficients of factors and the output gap are not correlated.

This leads to the next intriguing question: since the provinces with high TFP can use the given resources to create higher output value than the provinces with lower TFP, is it possible to maximize the country’s output value by allocating all the resources in a country to the provinces with the highest TFP? Allocating more factor resources to provinces with high TFP within a certain range can indeed increase the GDP of a country. However, if excessive production factor resources are allowed to flow into a certain region, the region will not be able to efficiently utilize the resources for production due to the limited machinery for production and insufficient management and organizational structure of enterprises [35–38]. Moreover, if all the factors of a country are supplied to the provinces with the highest TFP, the factors must be oversupplied, causing factor prices to fall. Rational factor suppliers inevitably transfer factors to other provinces with higher factor prices. Therefore, it’s absurd to allocate all the factors to the province with the highest TFP. When building the model, it is calculated that the optimal allocation share of production factors of a province under competitive equilibrium is: $\frac{K_{i}}{K}=\frac{s_{i}\beta_{Ki}}{{\bar{\beta }}_{K}}{\tilde{\gamma }}_{Ki}$ (taking capital as an example). The calculation result demonstrates the factor allocation share that maximizes the output value in the presence of factor price distortions.

## Conclusion

This paper constructs a competition model with factor price distortions, and uses this model to analyze the resource misallocation among provinces in China. Most literature only takes the two factors of capital and labor into consideration, but this paper holds that land is also an essential factor in China and incorporates it into the analysis. Panel data from 2004 to 2020 for 275 cities covering 31 provinces in China is adopted, which is more robust and reliable than provincial-level data used in other studies. This paper draws the following conclusions through empirical analysis. First, there is a huge gap in TFP among different provinces in China, and the value gradually decreases from the east to the west. Second, the costs of factors in east and middle China are higher than those in west China. In addition, the cost distortions between provinces cause a loss of about 6% of China’s output value. Third, China’s economic growth mainly depends on the increase of factor input (accounting for about 65%) and the TFP growth (accounting for about 30%). The improvement in factor price distortion contributes has no significant effect on the growth. Fourth, the price of land for production in China is severely distorted, indicating that the price of land sold by the government is far lower than the value created by enterprises using the land. Finally, the share of factors used in provinces with high TFP is gradually increasing, suggesting that in China, the production factors are flowing from provinces with low TFP to provinces with high TFP, and the resource misallocation has been improved.

This paper puts forward the following policy recommendations based on the conclusions drawn. Firstly, reduce the degree of distortion in factor markets. The price distortion of factors of production can be reduced by allowing the power of supply and demand to adjust factor prices between different regions. In addition, policy barriers that impede the flow of factors of production need to be reduced to allow factors to flow freely between regions of China. Second, the central government should formulate regional economic development policies to balance the productivity differences between the east, middle and west regions. The government can consider giving financial subsidies and policy assistance to industries in the western region and use a combination of measures to allow the productivity levels in the western region to catch up with those in the eastern and central regions. Thirdly, improve total factor productivity and the efficiency of resource allocation. China’s future economic growth can rely on more than just the increase in factor inputs. Improving total factor productivity and resource allocation efficiency is the only way to ensure sustainable growth of China’s economy. Fourth, establish a freely traded market of land for production. Currently, the price of land sold by local governments to productive enterprises needs to be more valued, resulting in a mismatch of resources for land. By establishing a freely traded land market, the price distortion of land can be eliminated.

There are also limitations in this paper. First, this paper only quantifies the degree of factor misallocation in each province, but does not analyze the causes. This is a research direction worth studying because only by finding out the causes of resource misallocation can we solve the problems. Second, the policies issued by the Chinese government enable the western region to obtain cheaper production factors, which leads to factor misallocation and loss of efficiency among regions. However, this study ignores that these policies may bring huge social benefits, such as narrowing the gap between the rich and the poor and maintaining the balance of development between regions. A certain degree of factor misallocation may be beneficial to the balanced development of society. The Chinese government is facing a trade-off between the loss caused by factor misallocation and the balanced development of society. Therefore, future research on misallocation should not only take output maximization as the only objective function, but also consider the potential social benefits resulting from misallocation.

## Supporting information

S1 File Appendix of the main text. It shows the detail derivation processes of Eqs (9), (14) and (15), which are also mentioned in the main text.
(DOCX)

S2 File Accessible channels of all original data used in this paper are showing in this file. (DOCX)

S3 File Original data used in the calculations of all figures and tables. It contains the original data of GDP, annual fixed asset investment, average salary, etc., and the source and acquisition way of the data are mentioned in S2 File.
(ZIP)

## References

[1] Shen Y, Yue S, Sun S, Guo M. Sustainable total factor productivity growth: The case of China. Journal of Cleaner Production. 2020;256:120727-. doi: 10.1016/j.jclepro.2020.120727

[2] Wang KL, Zhao B, Fan TZ, Zhang JN. Economic Growth Targets and Carbon Emissions: Evidence from China. International Journal of Environmental Research and Public Health. 2022;19(13). doi: 10.3390/ijerph19138053 35805709 PMC9265443

[3] Zhu Y, Liang DP, Liu TS, Song YZ. The impact of production factor distortion on total factor energy productivity: insight from China’s region level. Environmental Science and Pollution Research. 2020;27(32):40715–31. doi: 10.1007/s11356-020-09791-0 32666465

[4] Cui Y, Lu C. Are China’s unit labour costs still competitive? A comparison with ASEAN countries. Asian-Pacific Economic Literature. 2018;32(1):59–76. doi: 10.1111/apel.12217

[5] Sun X, Ping ZB, Dong ZF, Chen KL, Zhu XD, Li BL, et al. Resources and environmental costs of China’s rapid economic growth: From the latest theoretic SEEA framework to modeling practice. Journal of Cleaner Production. 2021;315:128126-. doi: 10.1016/j.jclepro.2021.128126

[6] Li X, Zhao X. Does low birth rate affect China’s total factor productivity? Economic Research-Ekonomska Istraživanja. 2022;35(1):2712–31. doi: 10.1080/1331677x.2021.1977671

[7] Ye YF, Chen SL, Li CN. Financial technology as a driver of poverty alleviation in China: Evidence from an innovative regression approach. Journal of Innovation & Knowledge. 2022;7(1). doi: 10.1016/j.jik.2022.100164

[8] Zhu F, Li Q, Yang S, Balezentis T. How ICT and R&D affect productivity? Firm level evidence for China. Ekonomska Istraživanja / Economic Research. 2021;34(35):1–19. doi: 10.1080/1331677X.2021.1875861

[9] Hsieh C-T, Klenow PJ. Misallocation and manufacturing TFP in China and India. The Quarterly journal of economics. 2009;124(4):1403–48. doi: 10.2139/ssrn.1442871

[10] Dollar D, Wei S-J. Das (Wasted) Kapital: Firm Ownership and Investment Efficiency in China. National Bureau of Economic Research Working Paper Series. 2007;No. 13103.

[11] Restuccia D, Rogerson R. The Causes and Costs of Misallocation. Journal of Economic Perspectives. 2017;31(3):151–74. doi: 10.1257/jep.31.3.151

[12] Wang YL, Lei XD, Yang F, Zhao N. Financial friction, resource misallocation and total factor productivity: theory and evidence from China. Journal of Applied Economics. 2021;24(1):393–408. doi: 10.1080/15140326.2021.1936836

[13] Adamopoulos T, Brandt L, Leight J, Restuccia D. Misallocation, Selection, and Productivity: A Quantitative Analysis With Panel Data From China. Econometrica. 2022;90(3):1261–82. doi: 10.3982/ecta16598

[14] Yang MA, Yang FX, Sun CW. Factor market distortion correction, resource reallocation and potential productivity gains: An empirical study on China’s heavy industry sector. Energy Economics. 2018;69:270–9. doi: 10.1016/j.eneco.2017.11.021

[15] Brandt L, Tombe T, Zhu XD. Factor market distortions across time, space and sectors in China. Review of Economic Dynamics. 2013;16(1):39–58. doi: 10.1016/j.red.2012.10.002

[16] Chen YW, Hu WM. Distortions, misallocation and losses: theory and application. China Economic Quarterly. 2011;10(4):1401–22. doi: 10.13821/j.cnki.ceq.2011.04.010 (in Chinese).

[17] Bun MJG, de Winter J. Capital and labor misallocation in the Netherlands. Journal of Productivity Analysis. 2022;57(1):93–113. doi: 10.1007/s11123-021-00622-z

[18] Hang J. Capacity utilization and the measurement of misallocation. Economics Letters. 2022;214. doi: 10.1016/j.econlet.2022.110410

[19] Tang LJ, Wang DY. Optimization of County-Level Land Resource Allocation through the Improvement of Allocation Efficiency from the Perspective of Sustainable Development. International Journal of Environmental Research and Public Health. 2018;15(12). doi: 10.3390/ijerph15122638 30477267 PMC6313350

[20] Jin WF, Zhou CS, Luo LJ. Impact of Land Input on Economic Growth at Different Stages of Development in Chinese Cities and Regions. Sustainability. 2018;10(8). doi: 10.3390/su10082847

[21] Bai CE, Hsieh CT, Song Z. Special Deals with Chinese Characteristics. Nber Macroeconomics Annual. 2020;34(1):341–79. doi: 10.1086/707189

[22] Bai Y, Jin K, Lu D. Misallocation under trade liberalization. *National Bureau of Economic Research*, 2019. https://www.nber.org/papers/w26188

[23] Restuccia D, Rogerson R. Policy distortions and aggregate productivity with heterogeneous establishments. Review of Economic Dynamics. 2008;11(4):707–20. doi: 10.1016/j.red.2008.05.002

[24] Restuccia D, Rogerson R. Misallocation and productivity. Review of Economic Dynamics. 2013;16(1). doi: 10.1016/j.red.2012.11.003

[25] Hopenhayn H. Job Turnover and Policy Evaluation: A General Equilibrium Analysis. Journal of Political Economy. 2016;101(5):915–38. doi: 10.1086/261909

[26] Gong L, Xie D. Factor mobility and dispersion in marginal products: A case on China. Frontiers of Economics in China. 2006;1:1–13. doi: 10.1007/s11459-005-0006-x

[27] Syrquin M. Resource reallocation and productivity growth. Economic Structure and Performance. 1984:75–101. doi: 10.1016/B978-0-12-680060-9.50011-8

[28] Duranton G, Turner MA. Urban growth and transportation. Review of Economic Studies. 2012;79(4):1407–40. Available from: https://www.cepr.org/active/publications/discussion_papers/dp.php?dpno=6633.

[29] Goldsmith RW. A perpetual inventory of national wealth. Studies in Income and Wealth, Volume 14: National Bureau of Economic Research; 1951. p. 5–73. https://www.nber.org/books-and-chapters/studies-income-and-wealth-volume-14/perpetual-inventory-national-wealth.

[30] Olley GS, Pakes A. The Dynamics of Productivity in the Telecommunications Equipment Industry. Econometrica. 1996;64(6):1263–97. doi: 10.3386/W3977

[31] Du WJ, Li MJ. The impact of land resource mismatch and land marketization on pollution emissions of industrial enterprises in China. Journal of Environmental Management. 2021;299. doi: 10.1016/j.jenvman.2021.113565 34419727

[32] Liu Y, Geng H. Regional Competition in China under the Price Distortion of Construction Land: A Study Based on a Two-regime Spatial Durbin Model. China & World Economy. 2019;27(4):104–26. doi: 10.1111/cwe.12288

[33] Tu F, Zou SL, Ding R. How Do Land Use Regulations Influence Industrial Land Prices? Evidence From China. International Journal of Strategic Property Management. 2021;25(1):76–89. doi: 10.3846/ijspm.2020.14051

[34] Lee MT, Suh I. Understanding the effects of Environment, Social, and Governance conduct on financial performance: Arguments for a process and integrated modelling approach. Sustainable Technology and Entrepreneurship. 2022;1(1):100004. doi: 10.1016/j.stae.2022.100004

[35] Chu SN, Song LG. Promoting Private Entrepreneurship for Deepening Market Reform in China: A Resource Allocation Perspective. China & World Economy. 2015;23(1):47–77. doi: 10.1111/cwe.12099

[36] Montoro-Sanchez A, Soriano DR. Human resource management and corporate entrepreneurship. International Journal of Manpower. 2011;32(1):6–13. doi: 10.1108/01437721111121198

[37] Ortigueira-Sánchez LC, Welsh DH, Stein WC. Innovation drivers for export performance. Sustainable Technology and Entrepreneurship. 2022;1(2):100013. doi: 10.1016/j.stae.2022.100013

[38] Torrent-Sellens J, Diaz-Chao A, Miro-Perez A-P, Sainz J. Towards the Tyrell corporation? Digitisation, firm-size and productivity divergence in Spain. Journal of Innovation & Knowledge. 2022;7(2):100185. doi: 10.1016/j.jik.2022.100185

