# Supplementary material for: Factor price distortion among regions in China and its influence on China’s economic growth
Source: PLoS One. 2023 Apr 10;18(4):e0284191. doi: 10.1371/journal.pone.0284191 (PMC10085039; doi:10.1371/journal.pone.0284191)
Supplement: S1 File — It shows the detail derivation processes of Eqs (9), (14) and (15), which are also mentioned in the main text. (DOCX) [file pone.0284191.s001.docx]

**Appendix**

**Appendix 1: Factor usage under competitive equilibrium**

The following is the derivation process of equation (9).

The equation (9) can be derived from equation (5) and equation (8), taking the capital usage $K_{i}$ in the province $i$ as an example:

$$\begin{matrix} K_{i}=\frac{\frac{\beta_{Ki}P_{i}Y_{i}}{(1+\tau_{Ki})P_{K}}}{\sum_{j=1}^{N} \frac{\beta_{Kj}P_{j}Y}{(1+\tau_{Kj})P_{K}}}K=\frac{\frac{\beta_{Ki}P_{i}Y_{i}}{(1+\tau_{Ki})}}{\sum_{j=1}^{N} \frac{\beta_{Kj}P_{j}Y}{(1+\tau_{Kj})}}K=\frac{\frac{\beta_{Ki}P_{i}Y/Y}{(1+\tau_{Ki})}}{\sum_{j=1}^{N} \frac{\beta_{Kj}P_{j}Y/Y}{(1+\tau_{Kj})}}K \\ =\frac{\left(s_{i}/\bar{\beta}_{K}\right)\beta_{Ki}\gamma_{Ki}}{\sum_{j=1}^{N} \left(sj/\bar{\beta}_{K}\right)\beta_{Kj}\gamma_{Kj}}K=\left(s_{i}/\bar{\beta}_{K}\right)\beta_{Ki}\cdot\frac{\gamma_{Ki}}{\sum_{j=1}^{N} \left(sj/\bar{\beta}_{K}\right)\beta_{Kj}\gamma_{Kj}}\cdot K=\frac{s_{i}\beta_{Ki}}{\bar{\beta}_{K}}\tilde{\gamma}_{Ki}K \end{matrix}$$

Similarly, $L_{i}$ and $M_{i}$ can be derived.

**Appendix 2: Analysis on the growth of output value**

The following is the derivation process of equation (14).

The equation is deduced as:

$$\frac{\partial\ln Y_{t}}{\partial\ln Y_{it}}=\frac{\partial Y_{t}}{\partial Y_{it}}\cdot\frac{1/Y_{t}}{1/Y_{it}}=P_{it}\cdot\frac{Y_{it}}{Y_{t}}=s_{it}$$

where $\frac{\partial Y_{t}}{\partial Y_{it}}=P_{it}$ can be calculated by equation :$Y=\sum_{i=1}^{N} P_{i}Y_{i}$.

The change in output value$\Delta\ln Y_{t}$ from time t to time t+1 can be calculated by:

$$\begin{matrix} \Delta\ln Y_{t}=\ln Y_{t+1}-\ln Y_{t} \\ \approx\sum_{i=1}^{N} \frac{\partial\ln Y_{t}}{\partial\ln Y_{it}}(\ln Y_{it+1}-\ln Y_{it}) \\ =\sum_{i=1}^{N} s_{it}\Delta\ln Y_{it} \\ =\sum_{i=1}^{N} s_{it}\Delta\ln TFP_{i}+\sum_{i=1}^{N} s_{it}\ln[\left(\frac{s_{it+1}}{s_{it}}\right)/\left(\frac{\bar{\beta}^{\beta_{Ki}}_{Kt+1}\bar{\beta}^{\beta_{Li}}_{Lt+1}\bar{\beta}^{\beta_{Mi}}_{Mt+1}}{\bar{\beta}^{\beta_{Ki}}_{Kt}\bar{\beta}^{\beta_{Li}}_{Lt}\bar{\beta}^{\beta_{Mi}}_{Mt}}\right)] \\ +\sum_{i=1}^{N} s_{it}\left[\beta_{Ki}\Delta\ln\tilde{\gamma}_{Ki}+\beta_{Li}\Delta\ln\tilde{\gamma}_{Li}+\beta_{Mi}\Delta\ln\tilde{\gamma}_{Mi}\right] \\ +\sum_{i=1}^{N} s_{it}\left[\beta_{Ki}\Delta\ln K+\beta_{Li}\Delta\ln L+\beta_{Mi}\Delta\ln M\right] \end{matrix}$$

Specifically, the second part can be calculated using the first derivative form of Taylor’s theorem where the change in a country’s output value is approximately equal to the sum of the changes in the output value of its provinces

**Appendix 3: Derivation of the national production function**

The following is the derivation process of equation (15).

Suppose each province $i$ produces intermediate products $Y_{i}$, and different provinces produce different types of intermediate products. The country uses the intermediate products of each province as inputs to produce an end product. The production function of the country is: $Y=\prod_{i=1}^{N} Y_{i}^{\theta_{i}}$ , where, $\sum\theta_{i}=1$ and $\theta_{i}$ is a positive constant. N indicates that there are N kinds of intermediate products. The intermediate products are general abstract concepts and can be referred to as ownership, regions, or industries in other documents. The minimized input cost of the end product: $\min\sum_{i=1}^{N} P_{i}Y_{i}\text{ s.t. }Y=\prod_{i=1}^{N} Y_{i}^{\theta_{i}}$. It can be solved by the first derivative: $P_{i}Y_{i}=\theta_{i}Y$ (assuming the price of the end products $P=1$). It can be seen from the calculation results that when the cost is minimized, $\theta_{i}$ is the output value $s_{i}$. Therefore, the production function of the country is derived as: $Y=\prod_{i=1}^{N} Y_{i}^{s_{i}}$.
