# Supplementary material for: Factor price distortion among regions in China and its influence on China’s economic growth
Source: PLoS One. 2023 Apr 10;18(4):e0284191. doi: 10.1371/journal.pone.0284191 (PMC10085039; doi:10.1371/journal.pone.0284191)
Supplement: S2 File — (DOCX) [file pone.0284191.s002.docx]

**Data Availability**

All data used in this paper are from the National Bureau of Statistics of China (NBS) and the China Statistical Yearbook from 2004 to 2020. The author confirm he did not have any special access to this data which other researchers would not have.

**Way 1:**

Get all data used in this paper, please visit: (National Bureau of Statistics of China)

<http://www.stats.gov.cn/sj/>

(Detail way to get the specific data from the National Bureau of Statistics:

First choose the “Data Center”, then choose the “Data Query”, next choose the frequency of data. This paper used annual frequency data, so choose “Annual Data”. At this point, the indicator menu will appear on the left side of the page. After that, the indicators menu will show on the left side. Please choose the indicators "GDP", "Price Index", "Labor force population", "Employment and wages", "Fixed asset investment and real estate", "Resident income" and "Built-up area", and set the time range from 2004 to 2020, download the data.)

**Way 2:**

The above data can also be collected from the China Statistical Yearbook from 2004 to 2020, published by the National Bureau of Statistics of China (NBS). To view the Statistical Yearbook online, please visit: (the China Statistical Yearbook)

<http://www.stats.gov.cn/sj/ndsj/>

(Detail way to get the specific data from the China Statistical Yearbook:

Just click on the year in the menu bar and the website will jump to the statistical yearbook for that year. This paper is based on the data from 2004 to 2020. There is no doubt that Way 1 and Way 2 get the same data finally.)
