# Supplementary figures and images for: Factor price distortion among regions in China and its influence on China’s economic growth
Source: PLoS One. 2023 Apr 10;18(4):e0284191. doi: 10.1371/journal.pone.0284191 (PMC10085039; doi:10.1371/journal.pone.0284191)

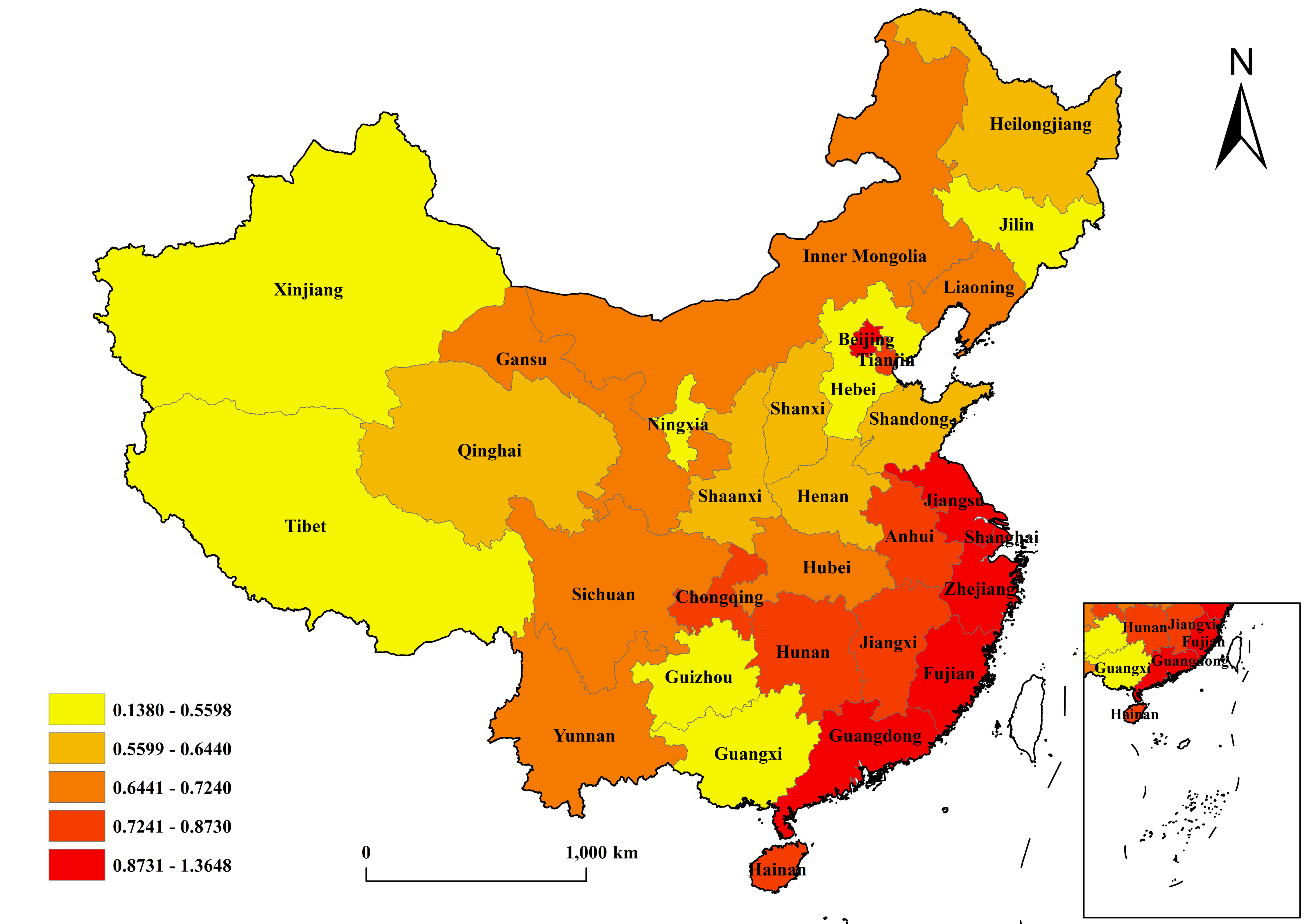

Supplement: S3 File — It contains the original data of GDP, annual fixed asset investment, average salary, etc., and the source and acquisition way of the data are mentioned in S2 File. (ZIP) [file pone.0284191.s003.zip › S3 file_raw data/Fig1-Logarithm of TFP of each province.tif]

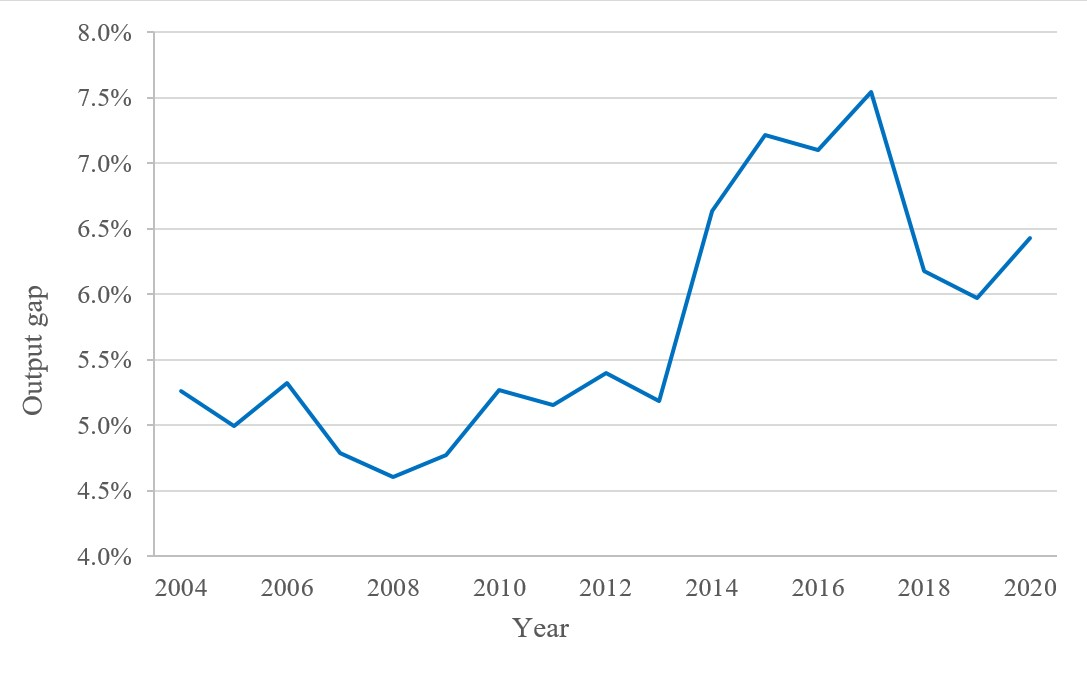

Supplement: S3 File — It contains the original data of GDP, annual fixed asset investment, average salary, etc., and the source and acquisition way of the data are mentioned in S2 File. (ZIP) [file pone.0284191.s003.zip › S3 file_raw data/Fig2-Output gap.tif]

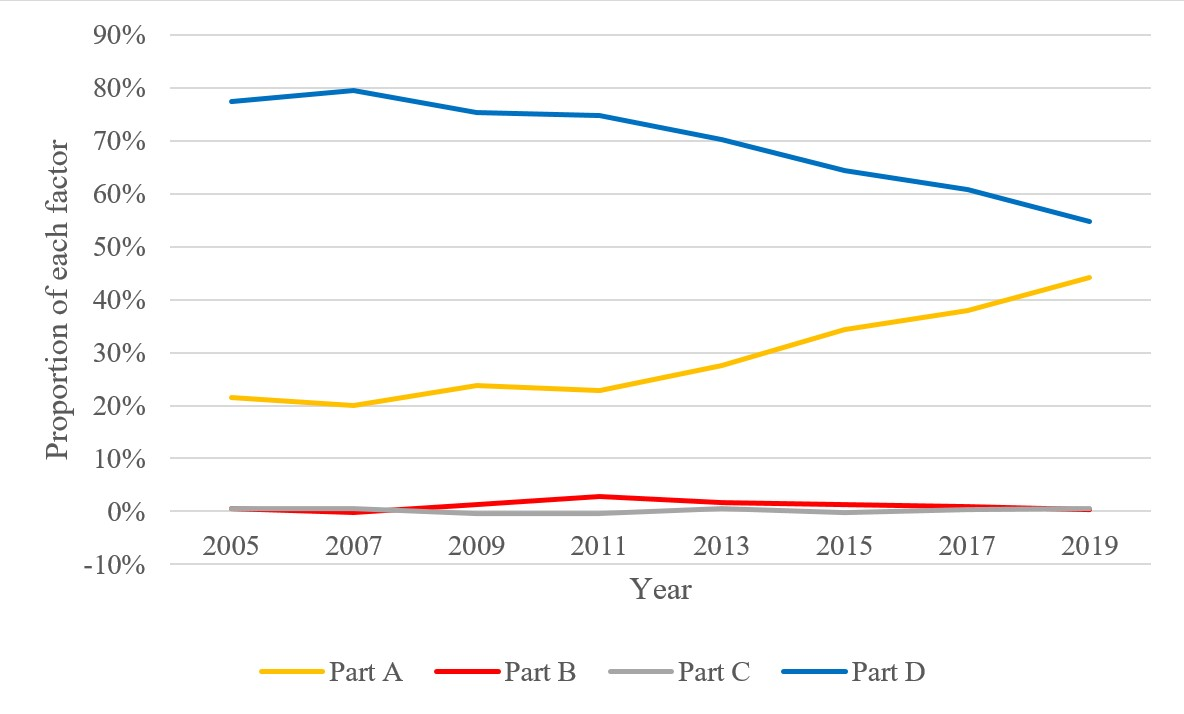

Supplement: S3 File — It contains the original data of GDP, annual fixed asset investment, average salary, etc., and the source and acquisition way of the data are mentioned in S2 File. (ZIP) [file pone.0284191.s003.zip › S3 file_raw data/Fig3-Analysis of the driving factors of economic growth.tif]

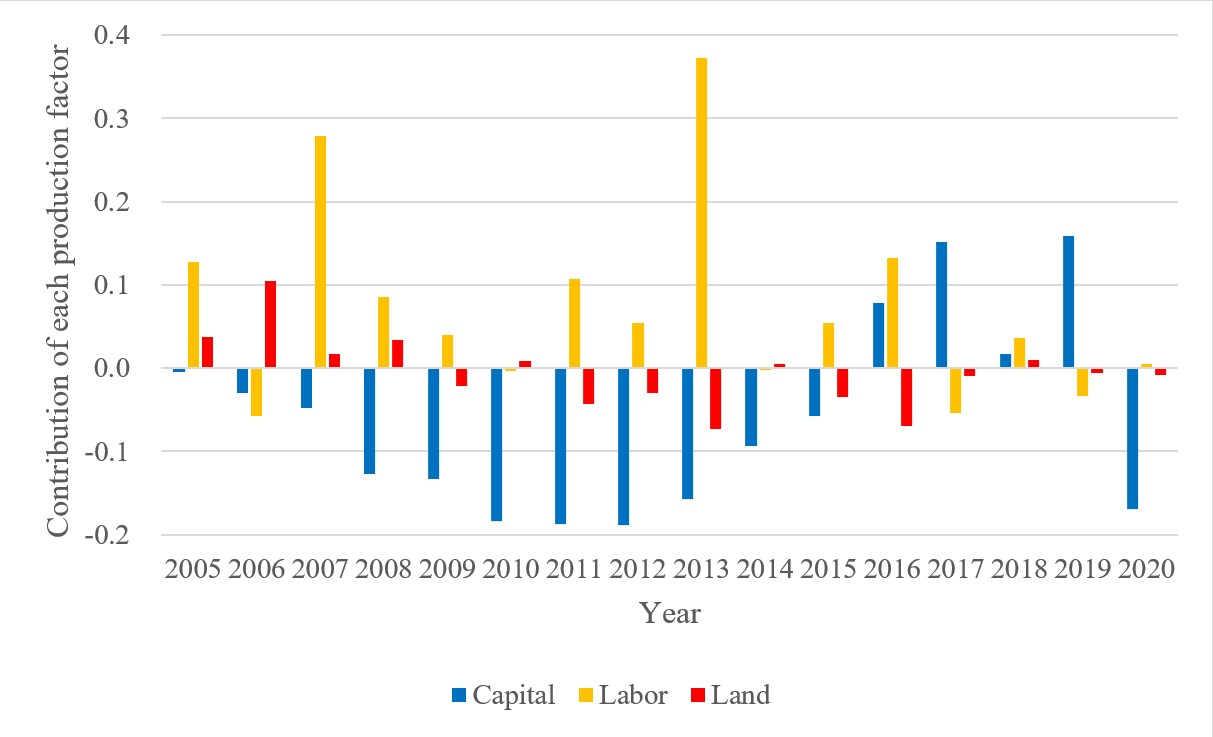

Supplement: S3 File — It contains the original data of GDP, annual fixed asset investment, average salary, etc., and the source and acquisition way of the data are mentioned in S2 File. (ZIP) [file pone.0284191.s003.zip › S3 file_raw data/Fig4-Contribution of changes in relative distortion coefficients to economic growth.tif]

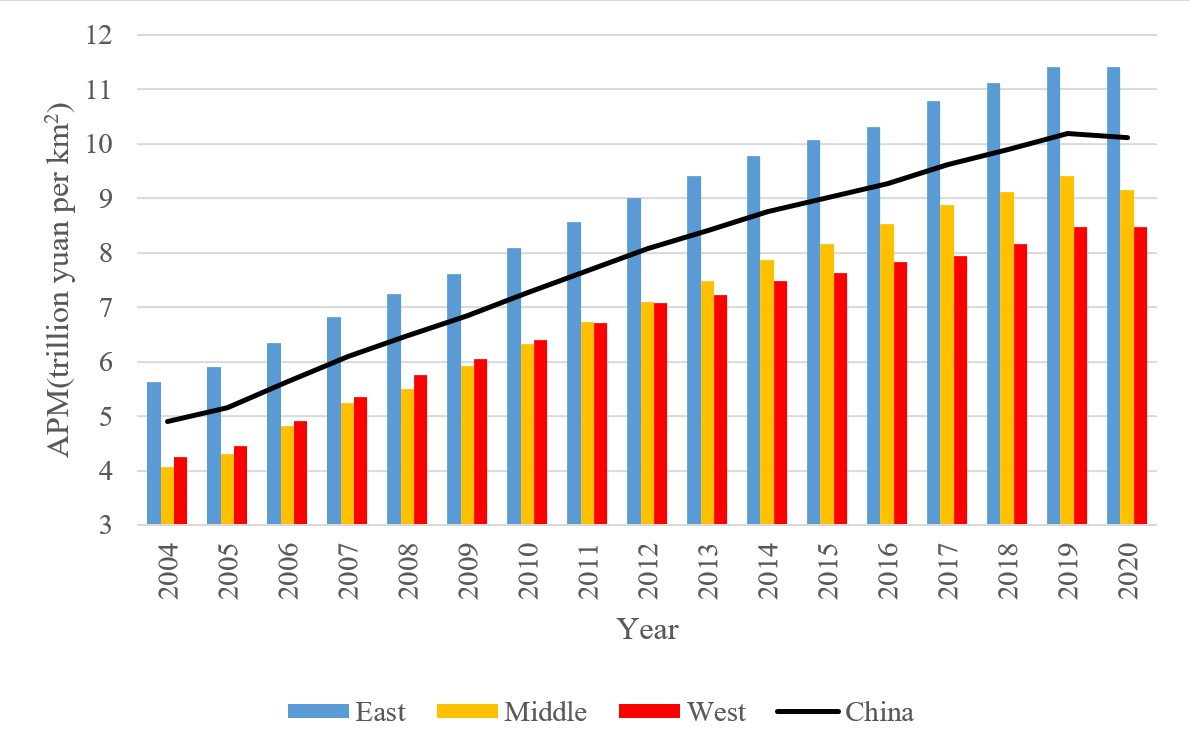

Supplement: S3 File — It contains the original data of GDP, annual fixed asset investment, average salary, etc., and the source and acquisition way of the data are mentioned in S2 File. (ZIP) [file pone.0284191.s003.zip › S3 file_raw data/Fig5-APM in different regions.tif]

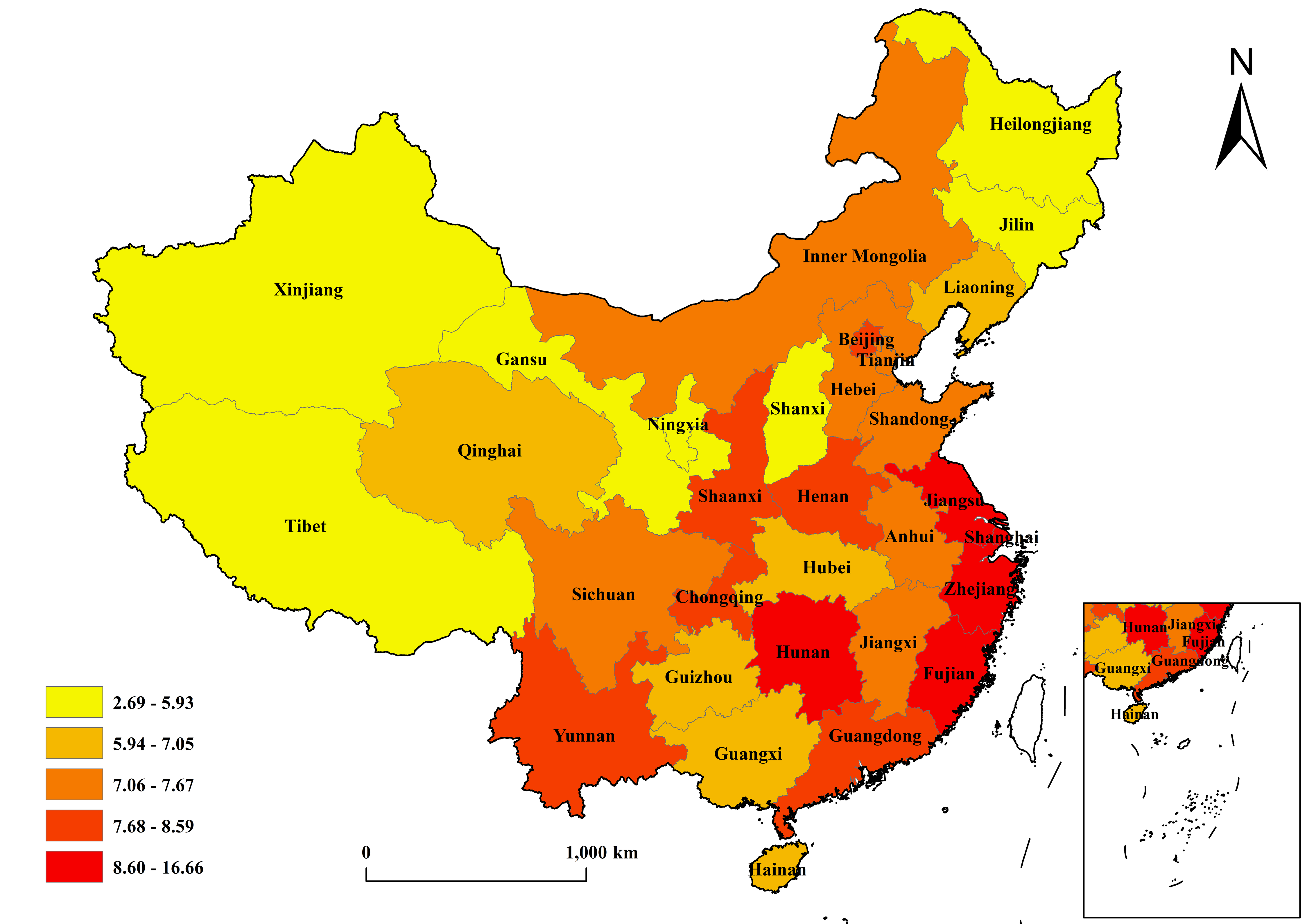

Supplement: S3 File — It contains the original data of GDP, annual fixed asset investment, average salary, etc., and the source and acquisition way of the data are mentioned in S2 File. (ZIP) [file pone.0284191.s003.zip › S3 file_raw data/Fig6- APM in different provinces.tif]
